# Manifestation of Huntington’s disease pathology in human induced pluripotent stem cell-derived neurons

**DOI:** 10.1186/s13024-016-0092-5

**Published:** 2016-04-14

**Authors:** Evgeny D. Nekrasov, Vladimir A. Vigont, Sergey A. Klyushnikov, Olga S. Lebedeva, Ekaterina M. Vassina, Alexandra N. Bogomazova, Ilya V. Chestkov, Tatiana A. Semashko, Elena Kiseleva, Lyubov A. Suldina, Pavel A. Bobrovsky, Olga A. Zimina, Maria A. Ryazantseva, Anton Yu. Skopin, Sergey N. Illarioshkin, Elena V. Kaznacheyeva, Maria A. Lagarkova, Sergey L. Kiselev

**Affiliations:** Vavilov Institute of General Genetics, Russian Academy of Sciences, Moscow, 119333 Russia; Institute of Cytology, Russian Academy of Sciences, St. Petersburg, 194064 Russia; Research Center of Neurology, Moscow, 125367 Russia; Scientific-Research Institute of Physical-Chemical Medicine, Moscow, 119435 Russia; Federal Research Center Institute of Cytology and Genetics SB RAS, Novosibirsk, 630090 Russia; Kazan State University, Kazan, 420008 Russia

**Keywords:** Huntington’s disease, Human induced pluripotent stem cells, Differentiation, GABAergic medium spiny neurons, Nuclear indentations, Store-operated calcium entry, Neurodegeneration, Aging, Neuroprotection

## Abstract

**Background:**

Huntington’s disease (HD) is an incurable hereditary neurodegenerative disorder, which manifests itself as a loss of GABAergic medium spiny (GABA MS) neurons in the striatum and caused by an expansion of the CAG repeat in exon 1 of the huntingtin gene. There is no cure for HD, existing pharmaceutical can only relieve its symptoms.

**Results:**

Here, induced pluripotent stem cells were established from patients with low CAG repeat expansion in the huntingtin gene, and were then efficiently differentiated into GABA MS-like neurons (GMSLNs) under defined culture conditions. The generated HD GMSLNs recapitulated disease pathology in vitro, as evidenced by mutant huntingtin protein aggregation, increased number of lysosomes/autophagosomes, nuclear indentations, and enhanced neuronal death during cell aging. Moreover, store-operated channel (SOC) currents were detected in the differentiated neurons, and enhanced calcium entry was reproducibly demonstrated in all HD GMSLNs genotypes. Additionally, the quinazoline derivative, EVP4593, reduced the number of lysosomes/autophagosomes and SOC currents in HD GMSLNs and exerted neuroprotective effects during cell aging.

**Conclusions:**

Our data is the first to demonstrate the direct link of nuclear morphology and SOC calcium deregulation to mutant huntingtin protein expression in iPSCs-derived neurons with disease-mimetic hallmarks, providing a valuable tool for identification of candidate anti-HD drugs. Our experiments demonstrated that EVP4593 may be a promising anti-HD drug.

**Electronic supplementary material:**

The online version of this article (doi:10.1186/s13024-016-0092-5) contains supplementary material, which is available to authorized users.

## Background

Huntington’s disease (HD) is an incurable autosomal dominant hereditary neurodegenerative disorder that typically manifests between 35–55 years of age. The worldwide prevalence of HD ranges from 0.5 (Japan) to 5–7 (Europe, USA, and Canada) cases per 100,000 individuals. HD is characterized by extensive neurodegeneration, primarily affecting GABAergic medium spiny (GABA MS) neurons in the striatum. Other brain regions also show substantial neuronal damage with disease progression [1].

HD is caused by an expansion of cytosine-adenine-guanine (CAG) repeats in the huntingtin gene (*HTT*) that leads to a pathological elongation of polyglutamine repeats in the huntingtin protein (HTT). The HD phenotype develops when the number of trinucleotide repeats in the *HTT* gene exceeds 36. The HTT protein normally interacts with hundreds of other proteins, and probably has multiple biological functions [2]. While wild-type HTT (wtHTT) and mutant HTT (mHTT) proteins are ubiquitously expressed in the brain, neurodegeneration in HD mainly affects the striatum. Furthermore, the neurotoxic actions of mHTT are significantly higher in the striatal neurons of aged vs. young animals [3]. Recent magnetic resonance imaging and positron emission tomography studies demonstrated that striatal atrophy in human HD patients is detectable even at 10 years before the onset of disease symptoms [4]. Nevertheless, the mechanism of mHTT action is not fully understood, and is often considered multifactorial.

HD pathology is linked to the deregulation of multiple cellular processes (e.g., autophagy [5], calcium homeostasis [6], and assorted mitochondrial functions [7, 8]), but the critical factors behind HD advance are still unknown. Various challenges complicate the deciphering of HD molecular mechanisms, including a limited access to human neurons, the complexity of the molecular mechanisms underlying HD pathology, and the lack of adequate animal models. The discovery of somatic cell reprogramming technology, as well as the development of differentiation protocols for human pluripotent stem cells (PSCs), have jointly engendered new disease models based on induced PSCs (iPSCs) derived from the somatic cells of patients with particular afflictions [9, 10]. Recently, a number of studies have reported that iPSCs derived from patients with HD (HD iPSCs) are useful for disease modeling and genetic correction assessment. In an initial study, HD iPSC-derived neurons with a high trinucleotide repeat number showed elevated caspase 3/7 activity during differentiation upon growth factor deprivation [11]. Interestingly, mHTT aggregates were detected in undifferentiated HD iPSCs upon proteasome inhibition or the extended (up to 40 weeks) maintenance of neural progenitor cells (NPCs) in vivo [12]. Later, HD iPSCs were used to reverse disease phenotype by a homologous recombination technique [13]. Nevertheless, HD iPSC lines carrying homozygous or heterozygous mutations with relatively low repeat numbers (i.e., 39–44) did not show elevated caspase levels, despite the suggestion of abnormal protein clearance [14]. Efficient generation of GABA MS-like neurons (GMSLNs) from ESCs [15] and HD iPSCs [16] was recently described. HD iPSC Consortium established and analyzed iPSC lines from three HD patients carrying various number of repeats (ranging from 60 up to 180). Similar to transgenic HD models, the disease phenotype was most pronounced in neural cell derivatives carrying 180 CAG repeats, although an increased cumulative risk of death was observed for all three HD genotypes (ranging from 60 to 180) relative to wild-type (WT) controls [17].

Despite the progress in iPSC-facilitated HD modeling, no significant advance in disease prevention or treatment has yet been reported partly because the number of relevant physiological models is limited. However, given that faulty calcium signaling reportedly contributes to disease progression in transgenic animal models, modified calcium signaling is now regarded as a major target of medical anti-HD drug development [6].

Here we report the derivation of iPSC lines from the skin fibroblasts of three human HD subjects carrying low-CAG repeat numbers (iPSHD11 (Q40), iPSHD22 (Q47), and iPSHD34 (Q42)), and describe an efficient protocol for the generation of enriched populations of GMSLNs. We utilized the established cell model to investigate disease manifestation and neuroprotective actions of candidate pharmacological compounds. Electrophysiology techniques were employed to measure calcium store-operated channel (SOC) currents in the differentiated neurons, and revealed enhanced SOC activity in HD GMSLNs. A candidate compound effectively decreased SOC-mediated calcium entry into HD GMSLNs, and protected aging HD neurons from cell death. Therefore, we propose that iPSC-differentiated HD GMSLNs, with their described pathophysiological abnormalities, provide an appropriate model for both fundamental and applied studies of neurodegeneration.

## Results

### Establishment and characterization of human HD iPSC lines

Cultures of primary dermal fibroblasts were established from skin biopsies of three female HD patients. All three patients gave their written informed consent for the use of sample material for research purposes. Fibroblasts from passages 1–3 were used to generate iPSCs, which were then characterized for pluripotency marker expression (Additional file 1: Figure S1A). A normal karyotype was confirmed by GTG-banding (Additional file 1: Figure S1B).

To validate HD iPSC pluripotency both in vitro and in vivo, we next investigated the differentiation capacity of the iPSCs into cells of all three germ layers and their ability to form teratomas (Additional file 1: Figure S1C, S1D). One iPSC line from each patient (namely, iPSHD11, iPSHD22, and iPSHD34) was selected for further evaluation. The number of CAG repeats in the selected HD iPSC lines was determined by Sanger sequencing. As a result, the iPSHD11 line carried 40/17 CAG repeats, while the iPSHD22 and iPSHD34 lines carried 47/16 and 42/27 repeats, respectively. Previously described WT human iPSC lines, endo-iPS12 and IPSRG2L [18], and one WT human embryonic stem cell (ESC) line, hESM01 [19], were used as controls. The number of CAG repeats in the WT lines was determined by RT-PCR analysis and did not exceed 26–29 (Additional file 1: Figure S1E).

### Differentiation of human PSCs into GMSLNs

**Fig. 1:**
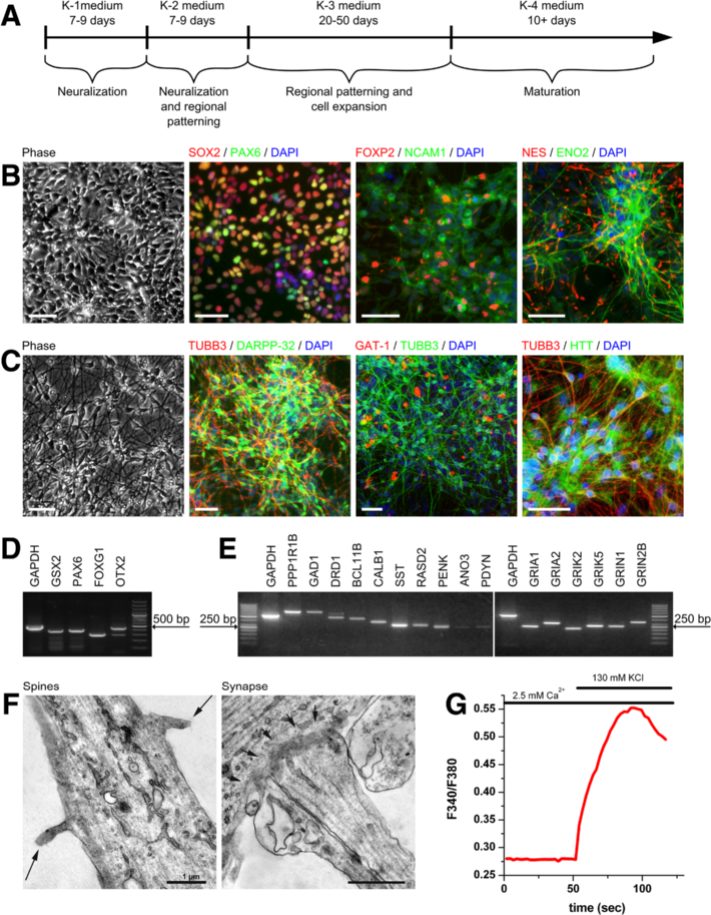
Characterization of GMSLNs differentiated from human PSCs. **a** Schematic representation of differentiation protocol. **b** Representative image of NPCs differentiated from hESM01 in phase contrast and immunostained for neuronal markers (SOX2, PAX6, FOXP2, NCAM1, ENO2, and Nestin), counterstained with DAPI. Scale bar, 50 μm. **c** Representative image of neurons differentiated from iPSHD34 in phase contrast and immunostained for DARPP-32, TUBB3, GAT1, and HTT (ab109115), counterstained with DAPI. Scale bar, 50 μm. **d** Representative RT-PCR analysis of NPCs differentiated from hESM01 showing expression of genes: *GSX2*, *PAX6*, *FOXG1*, and *OTX2*. **e** Representative RT-PCR analysis of neurons differentiated from endo-iPS12 showing expression of genes: *PPP1R1B*, *GAD1*, *DRD1*, *BCL11B*, *CALB1*, *SST, RASD2*, *PENK*, *ANO3*, *PDYN*, *GRIA1*, *GRIA2*, *GRIK2*, *GRIK5*, *GRIN1*, and *GRIN2B*. **f** Representative microphotographs of neurons differentiated from hESM01 acquired via TEM showing dendritic spines and synapse formation. **g** Cytosolic calcium cation levels in neurons differentiated from iPSHD22 in response to depolarization of the plasma membrane with potassium chloride (KCl). Cation levels were monitored by ratiometric Fura-2 imaging. Horizontal lines on the top of the graph indicate the time of application of 2.5 mM Ca^2+^ or 130 mM KCl into the culture medium

Human PSCs were maintained in mTeSR1 medium and cultured on a Matrigel™ matrix. A four-step protocol was developed to differentiate the PSCs into GMSLNs (Fig. 1a). First, the PSCs were directed toward differentiation into primitive neuroepithelial cells by using Noggin, SB431542, and dorsomorphin. After 7–9 days of culture, cell differentiation into primitive neuroepithelial cells was maintained via use of Noggin, and cell differentiation into lateral ganglionic eminence progenitors was initiated via use of purmorphamine [15]. This second differentiation step also required 7–9 days. Next, neural rosettes were mechanically replated to separate NPCs from other cell types, and the NPCs were expanded by using accutase passaging. NPCs were passaged up to four times (i.e., up to 50 days), frozen, or used directly for terminal differentiation.

Characterization of the NPCs using immunocytochemical (ICC) and RT-PCR analyses showed that the cells expressed *SOX2*, *PAX6*, *FOXP2*, *NCAM1*, *ENO2*, *Nestin*, *GSX2*, *FOXG1*, and *OTX2*, which are all expressed in developing striatum [20] (Fig. 1b, d). Following the initial differentiation procedure, the NPCs were further differentiated with brain-derived neurotrophic factor for ≥10 days for maturation into GMSLNs. ICC demonstrated that up to 93 ± 5 % of the cells specifically expressed TUBB3, a neuron-selective marker, while up to 79 ± 2 % of the TUBB3-positive cells specifically expressed DARPP-32, a GABA MS neurons marker [21] (Fig. 1c). The NPC-differentiated neurons also expressed the synaptic GABA transporter, GAT1, which removes GABA from the synaptic cleft and is indicative of synapse formation. Almost all of the differentiated HD and WT GMSLNs were positive for HTT (Fig. 1c).

The nucleus accumbens of the ventral striatum is represented with almost 95 % of GABA MS neurons therefore for additional characterization of the GMSLNs we accessed transcriptome databases [22, 23] and identified a group of genes co-expressed in this is region of striatum, but virtually absent from most other brain regions. GMSLNs differentiated from PSCs expressed all identified genes, namely *PPP1R1B, GAD1, DRD1, BCL11B, CALB1, SST, RASD2, PENK, ANO3, PDYN* (Fig. 1e).

Next, the cultivation medium of the terminally differentiated GMSLNs was withdrawn 30 min after incubation with cells and subjected to high-performance liquid chromatography analysis for GABA secretion. Consequently, the average GABA concentration was 508 ± 162 μM. We did not find any reproducible differences between normal and “diseased” samples. Transmission electron microscopy (TEM) analysis of differentiated GMSLNs demonstrated that the neurons had dendritic spines and were capable of forming synapses (Fig. 1f). The functional properties of the differentiated GMSLNs were then assessed in vitro by their response to potassium-mediated membrane depolarization. After depolarization, a significant calcium influx was detected by Fura-2 imaging (Fig. 1g). Collectively, these data confirm that our differentiation protocol generates neuronal cultures enriched in cell population with morphological and functional properties of GABA MS neurons.

### Recapitulation of disease pathology phenotype in the HD neuron model

To elucidate specific differences in the ability of pathological vs. normal PSCs to differentiate into neurons, we examined proliferation rates, NPC forming capacity, and the relative amount of DARPP-32 positive neurons in GMSLN cultures generated from the HD and the WT PSC lines. No significant differences were observed between the HD and WT cells concerning proliferation rates, NPC formation, or the proportion of DARPP-32 positive neurons in the last step of differentiation (Additional file 1: Figure S1F, S2A).

**Fig. 2:**
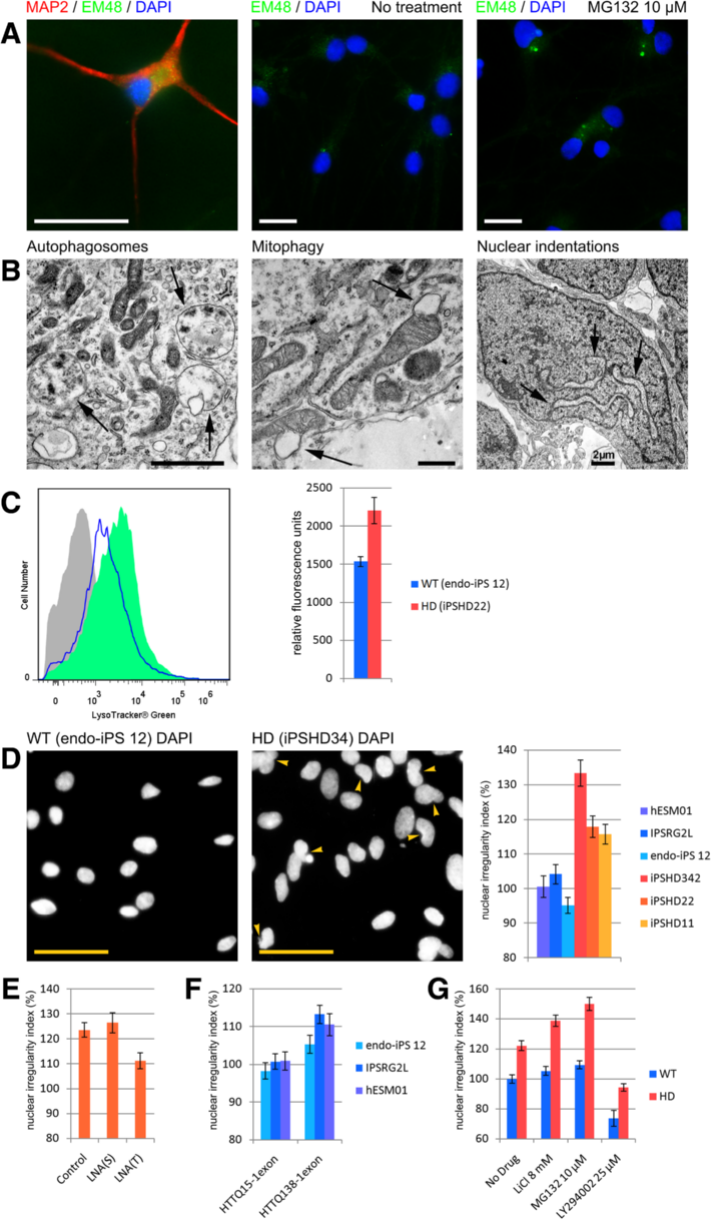
Phenotypic differences between HD and WT GMSLNs. **a** Representative microphotographs of 1) neurons differentiated from iPSHD22 cells, immunostained for microtubule-associated protein 2 (MAP2, *red*) and HTT (EM48 antibody, *green*), and counterstained with DAPI (*blue*); and 2) neurons differentiated from iPSHD22 cells, immunostained for HTT (EM48 antibody, *green*), and counterstained with DAPI (*blue*) following a 24 h incubation with or without 10 μM MG132. Scale bar, 20 μm. **b** Representative microphotograph of neurons differentiated from iPSHD22 cells acquired via TEM. Micrographs show high lysosomal and autophagosomal content, mitophagy, and nuclear envelope indentation. **c** Representative FC analysis of live neurons stained with LysoTracker Green; HD (*green*), WT (*blue*), and without staining (*grey*). The bar plot demonstrates median fluorescence intensity from three independent experiments. **d** Representative microphotographs of terminally differentiated WT and HD neurons stained with DAPI demonstrating nuclear indentations. Scale bar, 50 μm. The bar plot demonstrates morphometric quantification of nuclear irregularity index using 786–1340 nuclei per data point. **e** The bar plot demonstrates morphometric quantification of nuclear irregularity index in cultures of iPSHD22 derived neurons 4 days after transfection with antisense oligonucleotides: LNA(T) – specifically knockdown mHTT, LNA(S) – scrambled oligonucleotide, Control – no transfection. 754–1405 nuclei were count per data point. **f** The bar plot demonstrates morphometric quantification of nuclear irregularity index in cultures of WT PSCs (endo-iPS12, IPSRG2L and hESM01) derived neurons 9 days after infection with lentiviral constructs HTTQ15-1exon and HTTQ138-1exon. 754–1405 nuclei were count per data point. **g** Bar plot of the average mean nuclear irregularity index of HD (iPSHD11, iPSHD22, and iPSHD34) and WT (endo-iPS12, IPSRG2L, and hESM01) neurons after treatment with the indicated drugs. Morphometric quantification was conducted by using 2785–4649 nuclei per data point

We further investigated the in vitro differences between normal and pathological cultures by confirming mHTT protein presence in the differentiated HD neurons. Intracellular inclusions of aggregated mHTT are a hallmark of HD, and are readily demonstrated by using the EM48 antibody against the accumulated protein [24]. ICC revealed EM48-positive inclusions in 6-month-old, mature HD neurons (Fig. 2a), but not in WT neurons (Additional file 1: Figure S2B). Application of the proteasome inhibitor, MG132, to 6-month-old neurons at a concentration of 10 μM for 24 h significantly increased the number of EM48-positive inclusions in HD cultures (Fig. 2a), but not in WT cultures (Additional file 1: Figure S2B).

It was recently reported that aggregated mHTT disrupts the nuclear envelope [25]. Therefore, we investigated the morphology of WT and HD GMSLNs at the subcellular level via TEM. TEM analysis revealed larger numbers of lysosomes and autophagosomes, increased evidence of mitophagy, and a significantly enhanced occurrence of nuclear envelope indentations in HD vs. WT neurons, affecting 85 ± 5 % vs. 58 ± 3 % of the respective cell population (*p* < 0.005) (Fig. 2b).

We next performed a flow cytometry (FC) assay on live LysoTracker Green DND-26-stained GMSLNs to confirm the increased lysosomal content in HD neurons (Fig. 2c, Additional file 1: Figure S2C, for details see Additional file 1). As a result, neurons derived from HD iPSCs vs. WT PSCs exhibited a significantly higher median lysosomal content (2203 ± 172 vs. 1539 ± 65 relative fluorescence units, respectively; *p* < 0.05) (Fig. 2c).

Nuclear indentations were clearly evident not only by TEM (Fig. 2b), but also by DAPI staining of HD GMSLNs (Fig. 2d). To quantitatively assess nuclear indentation events, we conducted a nuclear morphometry analysis. This method has been successfully used in the study and diagnosis of various human pathologies [26, 27]. Here, we found a significantly higher mean nuclear irregularity index in HD neurons (116 ± 3 %, 118 ± 3 %, and 133 ± 4 % for iPSHD11, iPSHD22, and iPSHD34 lines, respectively; average = 122 ± 3 %) than in WT neurons (95 ± 2 %, 104 ± 3 %, and 101 ± 3 % for endo-iPS12, IPSRG2L, and hESM01 lines, respectively; average = 100 ± 3 %; *p* < 0.05) (Fig. 2d, g). A comparison of HD and WT fibroblasts or iPSCs revealed no significant differences in the nuclear irregularity index, suggesting that nuclear envelope impairment was selective for neurons undergoing further cell damage and death (Additional file 1: Figure S3A, S3B).

To verify that observed nuclear indentations were caused by endogenous mHTT we performed an allele-specific knockdown of mHTT using modified antisense oligonucleotides LNA(T) and LNA(S) described earlier [28] to block protein synthesis. It was nearly impossible to separate by electrophoresis normal and mutant forms of HTT with just a few amino acids difference, thus for these experiments we used fibroblasts and neurons from the cell line with a greatest difference in the number of repeats iPSHD22 (Q47/16). Transfection of parental fibroblasts (data not shown) or iPSHD22 derived neurons with LNA(T) but not with scrambled LNA(S) resulted in the significant reduction of mHTT at day 4 (Additional file 1: Figure S3C). We used transfected neurons to analyze nuclear indentations. We observed significant reduction of mean nuclear irregularity index in pathological neurons transfected with LNA(T) compared to LNA(S) or cells without treatment (*p* < 0.05) (Fig. 2e). To prove additionally that mHTT overexpression leads to the nuclear impairment in any normal neurons we introduced lentiviral constructs containing first exon of mutant huntigtin (HTTQ138-1exon) or normal huntingtin (HTTQ15-1exon) described earlier [29] into WT PSCs derived neurons. Protein overexpression (Additional file 1: Figure S3D) and nuclear irregularity index was measured 9 days after infection. We found that nuclear irregularity index was significantly higher in cultures of WT neurons infected with HTTQ138-1exon compared to WT neurons infected with HTTQ15-1exon constructs (*p* < 0.05) (Fig. 2f). These experiments confirm that observed nuclear indentations are caused by the presence of mHTT.

To globally assess the state of the nuclear envelope, we evaluated HD neurons for the distribution of lamin A, which provides mechanical strength to the envelope, and lamin B1, which contributes to envelope integrity [30]. No abnormalities were observed in either gross nuclear envelope morphology or lamin distribution (Additional file 1: Figure S3E).

We next investigated the nature of the nuclear indentations by evaluating the ability of MG132, a proteasomal inhibitor, and lithium chloride (LiCl), an autophagy inducer, to modify nuclear architecture. First, we explored whether MG132 could enhance the HD phenotype by promoting mHTT aggregation. Incubation of neurons with MG132 significantly increased the mean nuclear irregularity index in HD but not WT neurons, with observed post-incubation values of 123 ± 3 %, 172 ± 4 %, and 155 ± 6 % for iPSHD11, iPSHD22, and iPSHD34 lines, respectively (average = 150 ± 4 %; *p* < 0.05 vs. untreated HD neurons) (Fig. 2g, Additional file 1: Figure S3F). Next we examined the effect of LiCl, which was suggested recently as a possible drug for the treatment of HD via its ability to enhance mHTT clearance [31]. LiCl acted similarly to MG132, and increased the mean nuclear irregularity index in HD neurons (observed values = 127 ± 3 %, 126 ± 3 %, and 163 ± 5 % for iPSHD11, iPSHD22, and iPSHD34 lines, respectively; average = 139 ± 4 %; *p* < 0.05) (Fig. 2g, Additional file 1: Figure S3F). Nonetheless, our findings suppose that even superior mHTT clearance may be insufficient to protect neurons from nuclear indentations. Recently, it was shown that the phosphoinositide 3-kinase (PI3K) inhibitor, LY294002, corrected similar changes in nuclear shape in Parkinson’s disease (PD) model neurons [26]. Notably, LY294002 significantly reduced the mean nuclear irregularity index in HD neurons to 94 ± 2 %, 102 ± 2 %, and 87 ± 3 % for iPSHD11, iPSHD22, and iPSHD34 lines, respectively (average = 94 ± 2 %; *p* < 0.005 vs. untreated cells); however, the actions of the agent were not specific for pathological neurons, because LY294002-treated WT neurons also showed a significant reduction in the nuclear index, with observed post-incubation values of 82 ± 2 %, 68 ± 8 %, and 71 ± 3 % for endo-iPS12, IPSRG2L, and hESM01 lines (average = 74 ± 5 %; *p* < 0.005) (Fig. 2g, Additional file 1: Figure S3F). Although PI3K signaling is involved in nuclear organization, the PI3K cascade is apparently not impaired by mHTT expression in HD neurons.

Nuclear architecture is important for cellular functions directly connected with cell specialization and signaling [32]. Hence, global changes in nuclear structure should be reflected in gene expression patterns, prompting us to investigate differences in gene expression between HD and WT GMSLNs. We performed a comparative microarray-based transcriptome analysis by using RNA samples isolated from the three HD and three WT lines of differentiated GMSLNs, and identified 183 upregulated and 52 downregulated genes in the HD neurons (Additional file 1: Table S1). Additionally, we conducted an enrichment analysis by using the GOrilla web-based application [33] and demonstrated upregulation of calcium-related pathways in HD neurons (Additional file 1: Figure S4).

Taken together, the findings presented above demonstrate that HD iPSC-derived GMSLNs recapitulate multiple HD phenotypic characteristics and proteasome inhibition enhances HD manifestation.

### Abnormal SOC-mediated calcium entry in human HD GMSLNs is rescued by EVP4593

The transcriptome data and all abovementioned disease characteristics (i.e., increased lysosomal/autophagosomal content and mitophagy, and abnormal nuclear ultrastructure) are tightly associated with calcium homeostasis, suggesting the possible regulatory role of the latter in disease control. In this regard, calcium influx through SOCs is an important and ubiquitous mechanism for cation entry into mammalian cells, including neurons. Augmented SOC-mediated calcium entry was previously demonstrated in transgenic human neuroblastoma cell-based models of HD [34, 35]. Therefore, we set out to examine changes in calcium SOC currents between human HD vs. WT GMSLNs via electrophysiological means.

**Fig. 3:**
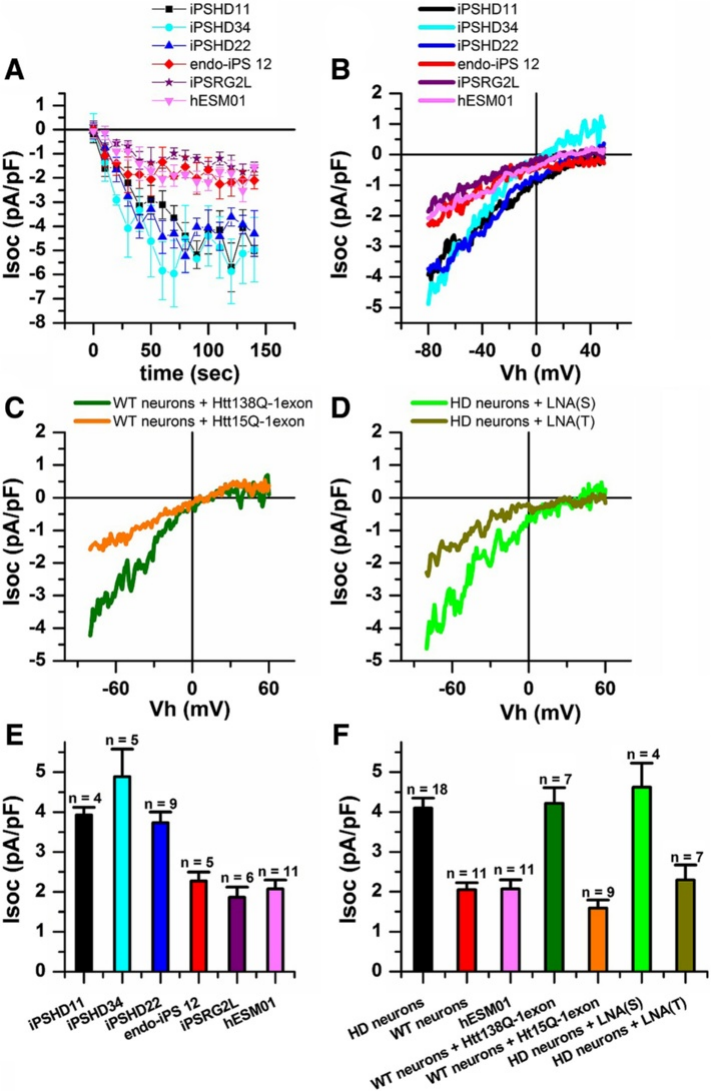
Enhanced SOC entry in GMSLNs differentiated from HD iPSCs. **a** SOC currents amplitudes recorded in whole-cell experiments as a function of time after application of Tg (1 μM) to HD GMSLNs differentiated from iPSHD11, iPSHD22, and iPSHD34 lines, or WT GMSLNs differentiated from IPSRG2L, endo-iPS12, and hESM01 lines. Current amplitudes for all groups were measured every 10 s at a test potential of –80 mV. Each plot shows mean ± SEM. **b** Average current-voltage (I-V) curves of currents evoked by passive depletion of calcium stores with Tg (1 μM) in GMSNLNs differentiated from HD iPSCs or WT PSCs. The I-V curves were recorded after full development of the SOC currents. Each trace represents the average of several experiments. **c**, **d** Average current-voltage (I-V) curves of currents evoked by passive depletion of calcium stores with Tg (1 μM). The I-V curves were recorded after full development of the SOC currents. Each trace represents the average of several experiments. **c** WT GMSNLNs infected with mutant HTTQ138-1exon or control HTTQ15-1exon. **d** In HD GMSNLNs transfected with LNA(T) specifically blocking mHTT expression or control LNA(S). **e** Average SOC current amplitude in GMSNLNs differentiated from HD iPSCs or WT PSCs. **f** Average amplitude of SOC currents in GMSLNs differentiated from HD iPSCs (*black symbols*), WT iPSCs (*red symbols*), WT ESCs (*light magenta symbols*); in WT GMSNLNs infected with mutant HTT138Q-1exon (*dark green symbols*) or control HTT15Q-1exon (*orange symbols*); in HD GMSNLNs transfected with LNA(T) (olive symbols) or control LNA(S) (*green symbols*). **e**, **f** For all groups, current amplitude was determined at a test potential of –80 mV and plotted as the mean ± SEM (n = number of single cell experiments)

To evoke a SOC-mediated calcium entry we used a standard and ubiquitous protocol with application of 1 μM Thapsigargin (Tg) [34, 36]. As a result, fully developed Tg-induced calcium currents exhibited mean amplitudes of 3.93 ± 0.19, 3.74 ± 0.27, and 4.88 ± 0.70 pA/pF in pathological iPSHD11, iPSHD22, and iPSHD34 cell lines, respectively. On the other hand, the Tg-induced currents exhibited mean amplitudes of only 1.86 ± 0.26, 2.27 ± 0.22, and 2.07 ± 0.22 pA/pF in WT IPSRG2L, endo-iPS12, and hESM01 lines, respectively (Fig. 3a, b, e). The average amplitudes were 4.10 ± 0.25 pA/pF in HD GMSLNs and 2.07 ± 0.22 pA/pF in WT GMSNLNs (Fig. 3f). Thus, the calcium current was ~2-fold higher in neurons derived from HD iPSCs relative to WT neurons derived from either iPSCs or ESCs (*p* < 0.05). Calcium imaging experiments based on Fura-2 fluorescence confirmed these results, and showed that SOC-mediated calcium entry was ~3-fold higher in HD GMSLNs (*p* < 0.05) (Additional file 1: Figure S5).

To prove that the observed calcium entry was caused by mHTT we measured SOC currents in the WT GMSNLNs infected with described above HTTQ138-1exon and HTTQ15-1exon lentiviral constructs (Additional file 1: Figure S3D). We found that the amplitude of SOC currents in mutant samples (HTTQ138-1exon) was significantly enhanced compared to control (HTTQ15-1exon) WT GMSNLNs, reaching a maximum of 4.22 ± 0.38 and of 1.59 ± 0.20 pA/pF, respectively (Fig. 3c, f). We also found that allele-specific knockdown of mutant huntingtin expression in HD GMSLNs using antisense oligonucleotide (Additional file 1: Figure S3C) decreased amplitude of abnormal SOC-mediated calcium entry from 4.62 ± 0.61 pA/pF in cells transfected with control LNA(S) to 2.29 ± 0.38 pA/pF in cells transfected with LNA(T) targeting mHTT (Fig. 3d, f). Therefore we could conclude that abnormal SOC-mediated calcium entry observed in HD human neurons is closely associated with the disease caused by mHTT.

**Fig. 4:**
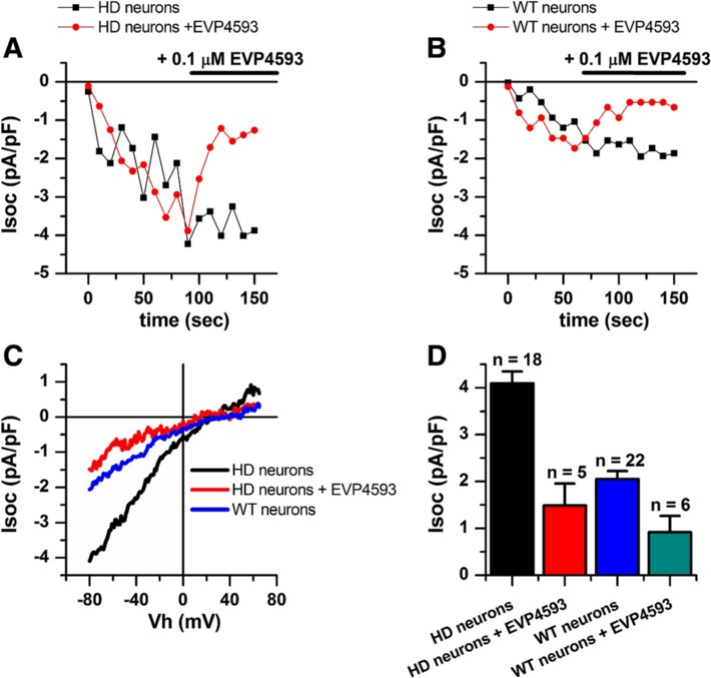
Reduction of SOC entry activity in HD GMSLNs by EVP4595. **a**, **b** Amplitude of Tg (1 μM)-induced currents in whole-cell experiments performed with (**a**) HD GMSLNs with (*red circles*) and without (*black squares*) EVP4593 (100 nM), or (b) WT GMSLNs with (*red circles*) and without (*black squares*) EVP4593 (100 nM). The 0 s data point corresponds to the time of Tg application. SOC amplitudes were measured at each ramp at a test potential of –80 mV. The time of EVP4593 application is shown above the plots. Each plot shows the results of one representative experiment. **c** Average I-V relationships for currents evoked by passive depletion of calcium stores with Tg (1 μM) in (1) HD GMSLNs after the currents reached the maximum (*black line*) and again after application of EVP4593 (100 nM) (*red line*), or (2) WT GMSLNs after the currents reached the maximum (blue line). Each trace represents the average of several experiments. **d** Average amplitude of SOCs in HD GMSLNs with (*red*) and without (*black*) EVP4593 (100 nM), or WT GMSLNs with (*teal*) and without (*blue*) EVP4593 (100 nM). The current amplitude for all groups was determined at a test potential of –80 mV and plotted as the mean ± SEM (n = number of single cell experiments)

The ability of the quinazoline derivative, EVP4593, to inhibit the SOC-mediated calcium entry pathway in transgenic HD animal models was demonstrated [34]. We therefore evaluated whether EVP4593 could similarly affect SOC-mediated calcium entry into HD GMSLNs. Application of EVP4593 at 100 nM to HD and WT GMSLNs reduced the amplitude of Tg-induced calcium currents from 4.10 ± 0.25 to 1.49 ± 0.47 pA/pF for HD neurons (Fig. 4a, c, d), and from 2.05 ± 0.18 to 0.92 ± 0.35 pA/pF for WT neurons (Fig. 4b, d). An analogous effect was observed with application of 3 μM EVP4593 (data not shown). These findings demonstrate the latent therapeutic capacity of EVP4593 to inhibit abnormal SOC currents, not only in HD neurons of transgenic models, but also in HD GMSLNs of human patients.

Altogether, the findings described above demonstrate significant deregulation of calcium transport in human HD neurons through SOCs within the plasma membrane. Notably, our results also show that HD GMSLNs respond to pharmacological agents targeted against these channels. Moreover, it must be emphasized that the calcium currents were recorded from HD GMSLNs obtained from different individuals by different approaches. The currents still showed similar characteristics, underscoring the validity and reproducibility of the iPSC-based model for HD studies and drug authentication.

### EVP4593 normalizes the number of lysosomes/autophagosomes in HD GMSLNs and rescues aging neurons from cell death

**Fig. 5:**
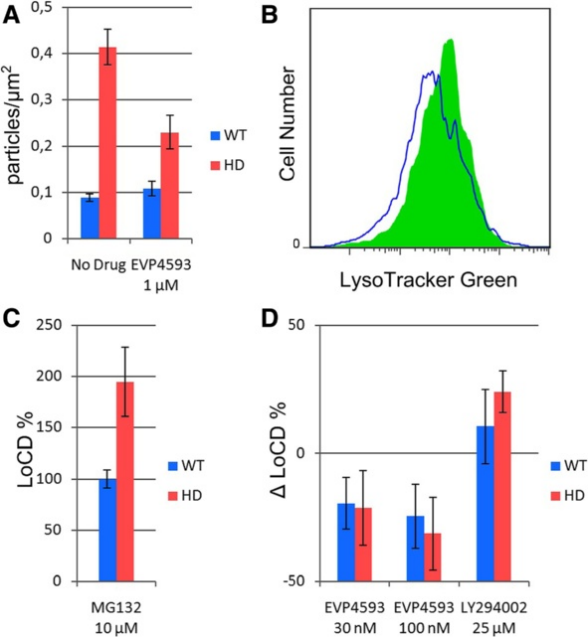
Protection of aging HD GMSNLNs from premature cell death. **a** The number of autophagosomes/lysosomes per μm^2^ in WT (hESM01) and HD (iPSHD22) GMSLNs after EVP4593 (1 μM) treatment for 14 h. The number of sections counted was 7-32 per bar. **b** Representative FC analysis of iPSHD22 MSNs stained with LysoTracker Green without treatment (green) and after 24 h incubation with EVP4593 100 nM (blue). **c** LoCD of WT and HD GMSLNs upon MG132-induced cell aging using 24 h 10 μM MG132 treatment. The WT value represents the mean LoCD for endo-iPS12-, IPSRG2L-, and hESM01-derived neurons, while the HD value represents the mean LoCD for iPSHD11-, iPSHD22-, and iPSHD34-derived neurons (*n* = 30). **d** EVP4593 safeguards against MG132-induced cell death in a dose-dependent manner. The WT value represents the mean ΔLoCD for endo-iPS12-, IPSRG2L-, and hESM01-derived neurons and the indicated drug, while the HD value represents the mean ΔLoCD for iPSHD11-, iPSHD22-, and iPSHD34-derived neurons and the indicated drug (*n* = 12). Each error bar represents the SEM

Normalization of calcium transport within neurons in response to EVP4593 is expected to reduce pathology manifestation. Therefore, we evaluated a number of lysosomes/autophagosomes in HD and WT neurons treated with EVP4593 using TEM. We found that incubation with EVP4593 reduced the number of lysosomes/autophagosomes in HD GMSLNs by almost two-fold (from 0.41 ± 0.04 to 0.23 ± 0.04; *p* < 0.05), while WT neurons were not affected (Fig. 5a). This observation was confirmed by examining lysosome content by FC analysis. The median fluorescence intensity was reduced by 34 ± 6 % in HD GMSLNs upon EVP4593 treatment (*p* < 0.05) (Fig. 5b). To rule out the possibility that proinflammatory signaling could be involved in EVP4593 response we measured NF-κB level in differentiated neurons. We did not find statistically significant differences between treated with EVP4593 and untreated samples (Additional file 1: Figure S6).

Because HD has a late onset, neuronal aging might possibly be involved in the cell death of HD GMSLNs. The efficiency of the proteostasis network declines with age, and this failure in protein homeostasis hypothetically underlies common age-related human disorders [37]. On these grounds, we established a novel cellular system by pharmacological means to mimic pathological neuronal cell death during aging. To do this, we applied the proteasome inhibitor, MG132, to WT and HD GMSLNs to model neuronal aging. We then measured the level of cell death (LoCD) in the neurons, and found that MG132-treated HD GMSNLNs were more susceptible to cell death (mean LoCD = 195 ± 34 %) than MG132-treated WT GMSLNs (mean LoCD = 100 ± 9 %; *p* < 0.01) (Fig. 5c).

Above, we demonstrated that LY294002 reduced nuclear indentations in HD neurons, while EVP4593 normalized SOC-mediated calcium entry and lysosomes/autophagosomes content. Here, we explored the ability of EVP4593 and LY294002 to rescue GMSLNs from MG132-induced cell death. HD and WT GMSLNs were treated with MG132 and the indicated drug, MG132 alone, drug alone, or solvent alone. The LoCD was measured in each case, and the differential actions of the indicated drugs against MG132-induced neuronal cell death (ΔLoCD) were calculated. EVP4593 was the only drug that significantly and dose-dependently reduced MG132-induced death of HD GMSLNs, with the highest efficacy at 100 nM (ΔLoCD = 31 ± 14 %; *p* < 0.05) (Fig. 5d).

## Discussion

Several PSC lines with mutations in the *HTT* gene have been described in earlier work, although the total number of cell lines carrying any particular number of trinucleotide repeats is still rather limited. Here, we report a study of HD iPSC lines with a low number of CAG repeats in the *HTT* gene that were established from the skin fibroblasts via lentiviral transduction. As controls, we included iPSCs established from healthy individuals [18], and also a previously described human ESC line [19] to omit possible influences of lentiviral integration.

The obtained pathological and control cells were next utilized to develop an efficient protocol based on defined culture medium for their differentiation into GMSLNs. Up to 80 % of the derived neurons expressed DARPP-32, a marker widely used for GABA MS neurons [12, 21]. The new protocol described in this paper has the clear advantage that only well-demarcated chemical components are employed for cell differentiation. Generally, our approach is similar to that reported in a previous study [15], but is adapted to human iPSCs maintained in mTeSR1 medium, and utilizes lower amounts of recombinant growth factors and chemical components.

We compared the cell growth and differentiation abilities of normal and pathological cell lines, and failed to observe any differences between the cells. These findings are consistent with another previously published report, where HD iPSCs with a low trinucleotide repeat number showed no abnormalities regarding proliferation or differentiation capacity [14]. Nevertheless, cells with a higher number of repeats still readily show a pathological phenotype [17]. Therefore, our investigation of disease pathology in HD vs. WT cells encompassed two main tasks: first, to uncover signs of disease manifestation in in vitro-cultured neurons with a low-CAG repeat number; and second, to establish a functional in vitro model reflecting neuronal cell loss during aging.

To accomplish these goals, we took advantage of TEM to assess the ultrastructure of GMSLNs before cell death (i.e., prior to evidence of pathology). This approach revealed multiple differences in neuronal ultrastructure between WT and HD cells, including an increased number of autophagosome/lysosome-like structures in the latter.

Enhanced lysosomal activity was detected in earlier studies upon exposure of cells to stress [14]. In our experiments we confirmed this finding in the absence of cell stress demonstrating 50 % higher lysosomal activity in HD GMSLNs compared with that in normal controls.

Additionally, our study is the first to identify increased levels of nuclear indentation in human HD iPSC-derived GMSLNs. Importantly, nuclear impairment was likewise demonstrated in earlier work with human postmortem brain slices of HD patients [38]. Although the molecular mechanisms particular to this phenomenon still remain to be investigated, nuclear morphometry clearly indicates a pathological phenotype that could be used to setup simple and scalable assays to examine the mechanisms involved.

Surprisingly, comparable changes in nuclear shape were observed in iPSC-derived NPCs carrying a *LRRK2* gene mutation, which results in the development of PD [26]. Incorrect protein folding and function are common in neurodegenerative disorders, suggesting that similar mechanisms might be involved in HD and PD pathology. LY294002 was previously shown to improve nuclear impairments in PD iPSC NPCs [26], and therefore, we reasoned that its administration might be helpful for the rescue of HD neurons. Nevertheless, while LY294002 dramatically reduced the nuclear indentation index in HD neurons, the compound also significantly impacted nuclear shape and induced dramatic impairments at the subcellular level in WT neurons. On the contrary, LiCl did not alter nuclear architecture in WT neurons but enhanced indentations in HD neurons. An increase in nuclear irregularity index suggests that other mechanisms than just impairment of the autophagic clearance of the excessive mHTT were responsible for nuclear envelope indentations in HD neurons.

We further investigated disease manifestation at the molecular level via transcriptome analysis, and found that nearly 200 genes changed their expression in HD vs. WT GMSLNs. Most of the identified genes are purportedly involved in calcium ion binding and calcium signaling, consistent with other findings of gene expression in HD neural cells [17, 39]. Moreover, we observed changes in the expression levels of transcripts identified in previous studies as contributing to pathology manifestation or mutant protein processing. For example, HD neurons showed an upregulation of *IFT57*, which could possibly trigger caspase-8 activation and neuronal death [40], as well as *ARHGEF6*, which enhances mHTT aggregation [41], and *IRS2*, which is required for mHTT clearance [42]. Surprisingly, our HD GMSLN cultures also overexpressed transcripts (i.e., *TIMP1* and *IER3* mRNAs, see Additional file 1: Table S1) that were recently proposed as HD biomarkers [43, 44].

We specifically aimed to disclose possible alterations in calcium homeostasis in our newly developed HD iPSC model. The role of calcium in HD pathogenesis has been extensively studied over the past decade [45]. Additionally, neuroblastoma cells expressing mHTT with 138 glutamine residues exhibit heightened SOC-mediated calcium entry pathway activity, and the small-molecule compound, EVP4593, which was initially found in a *Drosophila* screen, can successfully normalize SOC-mediated calcium entry [34].

It is generally accepted that transgenic HD models with low trinucleotide repeat numbers are not suitable for disease studies due to the lack of a functional phenotype. Meanwhile, HD manifestation is observed in humans with CAG repeat numbers reaching 36 and more. Therefore, we utilized iPSCs with different numbers of CAG repeats to optimize our results, and recorded SOC currents in GMSLNs differentiated independently from various HD and WT cell lines. Subsequently, HD neurons carrying 40–47 glutamine residues reproducibly exhibited 2-fold higher SOC currents relative to WT neurons harboring less than 29 repeats, and the elevated SOCs activity was accompanied by lysosomal accumulation in HD neurons. This is important because lysosomes share calcium storage functions with the endoplasmic reticulum, and lysosomal calcium can be released via transient receptor potential family channels [46]. Importantly, we failed to observe any differences between neurons differentiated from iPSCs or ESCs at the level of our present analyses, thus supporting the hypothesis that cell reprogramming introduces no significant epigenetic changes into final cell characteristics. Furthermore, the previously described quinazoline-derived compound, EVP4593, normalized calcium homeostasis and SOC currents in all patient-specific samples of pathological neurons, demonstrating its potential therapeutic utility. Additionally, we found that EVP4593 normalized lysosomes/autophagosomes content in HD GMSLNs indicating that the abnormalities in these systems are possibly mediated by Ca^2+^ dyshomeostasis rather than by autophagy impairment.

We next assessed the capacity of EVP4593 to defend aged HD neurons against MG132-induced neurotoxicity. HD has a late onset, and accordingly, neuronal cell loss is not generally observed in established HD models during their typically short time in culture (2–4 months). The proteasome is a major proteolytic system in mammalian cells, and carries out normal protein degradation as well as degradation of abnormal proteins that tend to accumulate during aging. Impairment of proteasome function is therefore tightly correlated with aging both in vivo and in vitro. In our model system, MG132-mediated proteasome inhibition enhanced the HD phenotype at the subcellular level, leading to increased formation of mHTT aggregates and nuclear indentations, and ultimately exacerbating neuronal cell death. However, EVP4593 significantly mitigated the MG132-induced cell death of HD GMSLNs.

Our findings together with the previously reported neuroprotective effects of EVP4593 in glutamate-toxicity assays [34], and the fact that EVP4593 acts at nanomolar concentrations, introduce this small molecule as a valuable new candidate for anti-HD drug development.

## Conclusions

In summary, we established an iPSC-derived cellular model of HD that showed disease manifestation in mature and aged neurons. We used three different patient-specific iPSC-derived cell lines to carefully evaluate the hypothesis that disrupted calcium signaling is behind HD pathology. Our results clearly support previous findings of calcium deregulation in HD, and suggest that this phenomenon may also underlie other HD symptoms. We also demonstrated enhanced SOCs activity in HD GMSLNs, and the promising neuroprotective properties of EVP4593 to reverse this process. Therefore, the iPSC model instituted herein may provide a useful platform for future fundamental studies of HD and drug development.

## Methods

### Cultivation of human PSCs and generation of iPSCs

iPSCs were generated as described in Additional file 1. The PSCs were cultured in mTeSR1 medium (Stemcell Technologies, Canada) on a Matrigel™ substrate (BD Biosciences, USA). Cells were passaged by using 1 mg/ml dispase neutral protease (Invitrogen, USA) and cryopreserved in mFreSR1 medium (Stemcell Technologies).

### Neuronal differentiation of human PSCs

Human PSCs were cultured in mTeSR1 medium on Matrigel™ until they reached 80–90 % confluency. The culture medium was then replaced with a 1:4 mixture of mTeSR1 and K-1 medium (see Additional file 1) for 2 days. The cells were maintained in K-1 medium for 5–7 days and in K-2 medium for the next 7–9 days. Next, the cells were transferred to K-3 medium, and neural rosettes were replated mechanically. NPCs were expanded by replating the cells with StemPro Accutase Cell Dissociation Reagent (Life Technologies, USA), followed by maintenance on Matrigel™ in K-3 medium until passage 4. At this time, the NPCs were transferred to K-4 medium for ≥10 days to generate mature neurons.

To prepare GMSLNs for electrophysiological recording and Fura-2 calcium imaging, differentiated neurons were plated onto 3 mm coverslips for 7–14 days prior to analysis. Twenty-four hours before analysis, the K-4 medium was exchanged with Neurobasal A Medium (Life Technologies) containing 3 % fetal bovine serum and 3 % B27 supplement (Life Technologies).

### Electrophysiological studies

Calcium currents were registered by using a whole-cell patch-clamp technique [47]. The measurements were taken with an Axopatch 200B Amplifier (Axon Instruments, USA). Microelectrode resistance was equivalent to 5–10 MOm; the series resistance was not compensated. Series resistance values were in range of 10–25 MOm and controlled all along the experiment. The currents were sampled at 5 kHz and filtered digitally at 500 Hz. pClamp6 software (Axon Instruments) was used for data acquisition and analysis. In all experiments, the holding potential was –40 mV. Membrane potential was periodically (every 5 s) dropped to –100 mV (for 30 ms), then gradually (1 mV/ms) increased to +100 mV and then returned to –40 mV. Measurements were made at 0.5-mV intervals. Registered currents were normalized to cellular capacitance (10–30 pF). The traces recorded before current activation were used as templates for leak subtraction. The pipette solution contained (in mM) 125 CsCl, 10 EGTA-Cs, 30 HEPES-Cs, 4.5 CaCl_2_, 1.5 MgCl_2_, 4 Na-ATP, pH 7.3. The extracellular solution contained (in mM) 140 NMDG-Asp, 10 BaCl_2_, 30 HEPES-Cs, 0.01 nifedipine, and 0.001 tetrodotoxin, pH 7.3. Currents were evoked by application of 1 μM Tg to the external solution.

### Quantitative analysis of cell nuclear morphology

Neurons were cultured in a 48-well plate in K-4 medium and treated with the indicated chemical compounds for 24 h before fixation. Cells were then fixed with 4 % paraformaldehyde (Sigma-Aldrich, USA) for 20 min at room temperature and stained with DAPI (Sigma-Aldrich). Images of 6–12 random fields in each well were obtained in a blind fashion using an Axiovert 40 CFL Fluorescence Microscope (Zeiss AG, Germany). The nuclear irregularity index was calculated using automatic processing of the images by the computer program. To identify cell nuclei the same hyperparameters were used for the whole experiment. The following equation was employed to calculate nuclear irregularity index: (nuclear perimeter^2^)/(4 × π × area). The nuclear irregularity index of WT neurons was taken to be 100 %. Calculations were performed with self-developed software, available on demand.

### Allele-specific mHTT knockdown using antisense oligonucleotides

Antisense oligonucleotides LNA(T) and LNA(S) described earlier [28] have the following sequences gcTgcTgcTgcTgcTgcTg and gcTatAccAgcGtcGtcAt, respectively. Locked nucleic acids are shown in capital. LNAs were synthesized and purified by DNA Synthesis LTD (Russia). Cells were plated on 48-well dishes at 100,000 cells/well. Stock solutions of oligonucleotides were heated at 65 °C for 5 min prior to use to dissolve any aggregates. Cells were transfected using TransIT®-LT1 Transfection Reagent (Mirus Bio, USA) according to the manufacturer’s instructions (1.5 μL of lipid was used for 100 pmol of oligos). mHTT knockdown was accessed by Western blotting four days after transfection.

### Quantitative analysis of cell death

Cells were cultured in K-4 medium in a 96-well black plates with clear flat bottom (Corning, USA). Next, cells were treated with chemical compounds for 24 h prior to analysis. Fluorescent assay MultiTox-Fluor Multiplex Cytotoxicity Assay (Promega, USA) was used to measure simultaneously the relative number of live (viability) and dead (cytotoxicity) cells in each well according to the manufacturer’s instructions. Fluorescence was detected by DTX 880 Multimode Microplate Reader (Beckman Coulter, USA). To evaluate the level of cell death (LoCD), the following equation was employed: ([cytotoxicity in a well with cells] − [cytotoxicity in a well without cells])/([viability in a well with cells] − [viability in a well without cells]). The LoCD of MG132-treated WT neurons was regarded as 100 %. To screen putative therapeutic compounds in the MG132-induced cell aging model, the following equation was used to determine differential action of the drug against MG132-induced neuronal cell death (ΔLoCD): ([LoCD in a well with 10 μM MG132 and drug] − [LoCD in a well with drug alone] − [LoCD in a well with 10 μM MG132 alone] + [LoCD in a well without MG132 or drug]).

### Microarray gene expression data

The microarray data was generated using HumanHT-12 v4 Expression BeadChip (Illumina, USA) according to manufacturer instructions and deposited in the Gene Expression Omnibus (GEO) database (accession number GSE77558). For details see Additional file 1.

### Statistical analysis

Each experiment was repeated at least three times. Quantifiable data are given as the mean ± the standard error of the mean. Comparisons of means were performed by using a one-tailed Student’s *t*-test or a one-tailed Welch’s *t*-test for unequal variances. In all cases, *p*-values <0.05 indicated statistically significant differences between means.

## Additional file

Additional file 1:
**Figure S1.** PSC lines characterization. **Figure S2.** HD and WT PSC derived neurons analysis. **Figure S3.** Nuclear indentations in HD PSC derived neurons. **Figure S4.** Classification of up-regulated genes in HD neurons compared to WT neurons with GOrilla tool by molecular function. **Figure S5.** Calcium entry evoked by store depletion is significantly increased in HD iPSCs derived neurons. **Figure S6.** NF-κB activity in PSCs derived neurons. **Table S1.** Differentially expressed genes in HD iPSCs derived neurons. (DOC 8958 kb)

## Abbreviations

iPSCs: Induced Pluripotent Stem Cells
ESCs: Embryonic Stem Cells
PSCs: Pluripotent Stem Cells
HD: Huntington’s Disease
NPCs: Neural Progenitor Cells
GABA MS: GABAergic Medium Spiny
GMSLNs: GABA MS-like neurons
SOC: Store-Operated Channel
LoCD: Level of Cell Death

## References

[1] Walker FO (2007). Huntington’s disease. Lancet.

[2] Tourette C, Li B, Bell R, O’Hare S, Kaltenbach LS, Mooney SD, Hughes RE (2014). A large scale huntingtin protein interaction network implicates RHO GTPase signaling pathways in huntington disease. J Biol Chem.

[3] Diguet E, Petit F, Escartin C, Cambon K, Bizat N, Dufour N, Hantraye P, Déglon N, Brouillet E (2009). Normal aging modulates the neurotoxicity of mutant huntingtin. PLoS One.

[4] Tabrizi SJ, Scahill RI, Owen G, Durr A, Leavitt BR, Roos RA (2013). Predictors of phenotypic progression and disease onset in premanifest and early-stage Huntington’s disease in the TRACK-HD study: Analysis of 36-month observational data. Lancet Neurol.

[5] Aki T, Funakoshi T, Unuma K, Uemura K (2013). Impairment of autophagy: from hereditary disorder to drug intoxication. Toxicology.

[6] Bezprozvanny I, Hayden MR (2004). Deranged neuronal calcium signaling and Huntington disease. Biochem Biophys Res Commun.

[7] Ayala-Peña S (2013). Role of oxidative DNA damage in mitochondrial dysfunction and Huntington’s disease pathogenesis. Free Radic Biol Med.

[8] Quintanilla RA, Jin YN, von Bernhardi R, Johnson GV (2013). Mitochondrial permeability transition pore induces mitochondria injury in Huntington disease. Mol Neurodegener.

[9] Sterneckert JL, Reinhardt P, Schöler HR (2014). Investigating human disease using stem cell models. Nat Rev Genet.

[10] Duan L, Bhattacharyya BJ, Belmadani A, Pan L, Miller RJ, Kessler JA (2014). Stem cell derived basal forebrain cholinergic neurons from Alzheimer’s disease patients are more susceptible to cell death. Mol Neurodegener.

[11] Zhang N, An MC, Montoro D, Ellerby LM (2010). Characterization of Human Huntington’s Disease Cell Model from Induced Pluripotent Stem Cells. PLoS Curr.

[12] Jeon I, Lee N, Li JY, Park IH, Park KS, Moon J (2012). Neuronal properties, in vivo effects, and pathology of a Huntington’s disease patient-derived induced pluripotent stem cells. Stem Cells.

[13] An MC, Zhang N, Scott G, Montoro D, Wittkop T, Mooney S (2012). Genetic correction of Huntington’s disease phenotypes in induced pluripotent stem cells. Cell Stem Cell.

[14] Camnasio S, Delli Carri A, Lombardo A, Grad I, Mariotti C, Castucci A, Rozell B, Lo Riso P, Castiglioni V, Zuccato C, Rochon C, Takashima Y, Diaferia G, Biunno I, Gellera C, Jaconi M, Smith A, Hovatta O, Naldini L, Di Donato S, Feki A, Cattaneo E (2012). The first reported generation of several induced pluripotent stem cell lines from homozygous and heterozygous Huntington’s disease patients demonstrates mutation related enhanced lysosomal activity. Neurobiol Dis.

[15] Ma L, Hu B, Liu Y, Vermilyea SC, Liu H, Gao L, Sun Y, Zhang X, Zhang SC (2012). Human embryonic stem cell-derived GABA neurons correct locomotion deficits in quinolinic acid-lesioned mice. Cell Stem Cell.

[16] Yao Y, Cui X, Al-Ramahi I, Sun X, Li B, Hou J, Difiglia M, Palacino J, Wu ZY, Ma L, Botas J, Lu B (2015). A striatal-enriched intronic GPCR modulates huntingtin levels and toxicity. Elife.

[17] The HD iPSC Consortium (2012). Induced pluripotent stem cells from patients with Huntington’s disease show CAG-repeat-expansion-associated phenotypes. Cell Stem Cell.

[18] Lagarkova MA, Shutova MV, Bogomazova AN, Vassina EM, Glazov EA, Zhang P (2010). Induction of pluripotency in human endothelial cells resets epigenetic profile on genome scale. Cell Cycle.

[19] Lagarkova MA, Volchkov PY, Lyakisheva AV, Philonenko ES, Kiselev SL (2006). Diverse epigenetic profile of novel human embryonic stem cell lines. Cell Cycle.

[20] Onorati M, Castiglioni V, Biasci D, Cesana E, Menon R, Vuono R, Talpo F, Goya RL, Lyons PA, Bulfamante GP, Muzio L, Martino G, Toselli M, Farina C, Barker RA, Biella G, Cattaneo E (2014). Molecular and functional definition of the developing human striatum. Nat Neurosci.

[21] Ouimet CC, Langley-Gullion KC, Greengard P (1998). Quantitative immunocytochemistry of DARPP-32-expressing neurons in the rat caudatoputamen. Brain Res.

[22] Hawrylycz MJ, Lein ES, Guillozet-Bongaarts AL, Shen EH, Ng L, Miller JA (2012). An anatomically comprehensive atlas of the adult human brain transcriptome. Nature.

[23] Le Carrour T, Assou S, Tondeur S, Lhermitte L, Lamb N, Reme T, Pantesco V, Hamamah S, Klein B, De Vos J (2010). Amazonia!: An Online Resource to Google and Visualize Public Human whole Genome Expression Data. Open Bioinforma J.

[24] Gutekunst CA, Li SH, Yi H, Mulroy JS, Kuemmerle S, Jones R, Rye D, Ferrante RJ, Hersch SM, Li XJ (1999). Nuclear and neuropil aggregates in Huntington’s disease: relationship to neuropathology. J Neurosci.

[25] Liu KY, Shyu YC, Barbaro BA, Lin YT, Chern Y, Thompson LM (2014). Disruption of the nuclear membrane by perinuclear inclusions of mutant huntingtin causes cell-cycle re-entry and striatal cell death in mouse and cell models of Huntington’s disease. Hum Mol Genet.

[26] Liu GH, Qu J, Suzuki K, Nivet E, Li M, Montserrat N (2012). Progressive degeneration of human neural stem cells caused by pathogenic LRRK2. Nature.

[27] Veltri RW, Isharwal S, Miller MC, Epstein JI, Partin AW (2010). Nuclear roundness variance predicts prostate cancer progression, metastasis, and death: A prospective evaluation with up to 25 years of follow-up after radical prostatectomy. Prostate.

[28] Gagnon KT, Pendergraff HM, Deleavey GF, Swayze EE, Potier P, Randolph J (2010). Allele-selective inhibition of mutant huntingtin expression with antisense oligonucleotides targeting the expanded CAG repeat. Biochemistry.

[29] Vigont VA, Zimina OA, Glushankova LN, Kolobkova JA, Ryazantseva MA, Mozhayeva GN, Kaznacheyeva EV (2014). STIM1 Protein Activates Store-Operated Calcium Channels in Cellular Model of Huntington’s Disease. Acta Naturae.

[30] Lammerding J, Fong LG, Ji JY, Reue K, Stewart CL, Young SG (2006). Lamins A and C but not lamin B1 regulate nuclear mechanics. J Biol Chem.

[31] Scheuing L, Chiu C, Liao H, Linares GR, Chuang D (2014). Preclinical and clinical investigations of mood stabilizers for Huntington’s disease: what have we learned?. Int J Biol Sci.

[32] Gdula MR, Poterlowicz K, Mardaryev AN, Sharov AA, Peng Y, Fessing MY (2013). Remodeling of three-dimensional organization of the nucleus during terminal keratinocyte differentiation in the epidermis. J Invest Dermatol.

[33] Eden E, Navon R, Steinfeld I, Lipson D, Yakhini Z (2009). GOrilla: a tool for discovery and visualization of enriched GO terms in ranked gene lists. BMC Bioinformatics.

[34] Wu J, Shih H-P, Vigont V, Hrdlicka L, Diggins L, Singh C (2011). Neuronal store-operated calcium entry pathway as a novel therapeutic target for Huntington’s disease treatment. Chem Biol.

[35] Glushankova LN, Zimina OA, Vigont VA, Mozhaeva GN, Bezprozvanny IB, Kaznacheeva EV (2010). Changes in the store-dependent calcium influx in a cellular model of Huntington’s disease. Dokl Biol Sci.

[36] Sharma S, Quintana A, Findlay GM, Mettlen M, Baust B, Jain M (2013). An siRNA screen for NFAT activation identifies septins as coordinators of store-operated Ca2+ entry. Nature.

[37] Morimoto RI, Cuervo AM (2014). Proteostasis and the aging proteome in health and disease. J Gerontol A Biol Sci Med Sci.

[38] Roos RA, Bots GT (1983). Nuclear membrane indentations in Huntington’s chorea. J Neurol Sci.

[39] Kalathur RK, Hernández-Prieto MA, Futschik ME (2012). Huntington’s Disease and its therapeutic target genes: A global functional profile based on the HD Research Crossroads database. BMC Neurology.

[40] Gervais FG, Singaraja R, Xanthoudakis S, Gutekunst CA, Leavitt BR, Metzler M (2002). Recruitment and activation of caspase-8 by the Huntingtin-interacting protein Hip-1 and a novel partner Hippi. Nat Cell Biol.

[41] Eriguchi M, Mizuta H, Luo S, Kuroda Y, Hara H, Rubinsztein DC (2010). alpha Pix enhances mutant huntingtin aggregation. J Neurol Sci.

[42] Yamamoto A, Cremona ML, Rothman JE (2006). Autophagy-mediated clearance of huntingtin aggregates triggered by the insulin-signaling pathway. J Cell Biol.

[43] Lorenzl S, Albers DS, LeWitt PA, Chirichigno JW, Hilgenberg SL, Cudkowicz ME (2003). Tissue inhibitors of matrix metalloproteinases are elevated in cerebrospinal fluid of neurodegenerative diseases. J Neurol Sci.

[44] Runne H, Kuhn A, Wild EJ, Pratyaksha W, Kristiansen M, Isaacs JD (2007). Analysis of potential transcriptomic biomarkers for Huntington’s disease in peripheral blood. Proc Natl Acad Sci U S A.

[45] Giacomello M, Oliveros JC, Naranjo JR, Carafoli E (2013). Neuronal Ca(2+) dyshomeostasis in Huntington disease. Prion.

[46] Lloyd-Evans E, Platt FM (2011). Lysosomal Ca(2+) homeostasis: role in pathogenesis of lysosomal storage diseases. Cell Calcium.

[47] Hamill OP, Sakmann B (1981). Multiple conductance states of single acetylcholine receptor channels in embryonic muscle cells. Nature.

