# Supplementary material for: Manifestation of Huntington’s disease pathology in human induced pluripotent stem cell-derived neurons
Source: Mol Neurodegener. 2016 Apr 14;11:27. doi: 10.1186/s13024-016-0092-5 (PMC4832474; doi:10.1186/s13024-016-0092-5)
Supplement: Additional file 1: — Figure S1. PSC lines characterization. Figure S2. HD and WT PSC derived neurons analysis. Figure S3. Nuclear indentations in HD PSC derived neurons. Figure S4. Classification of up-regulated genes in HD neurons compared to WT neurons with GOrilla tool by molecular function. Figure S5. Calcium entry evoked by store depletion is significantly increased in HD iPSCs derived neurons. Figure S6. NF-κB activity in PSCs derived neurons. Table S1. Differentially expressed genes in HD iPSCs derived neurons. (DOC 8958 kb) [file 13024_2016_92_MOESM1_ESM.doc]

**Supplemental Information**

**Inventory of Supplemental Information**

Figure S1. **PSC lines characterization.**

Figure S2. **HD and WT PSC derived neurons analysis.**

Figure S3. **Nuclear indentations in HD PSC derived neurons.**

Figure S4. **Classification of up-regulated genes in HD neurons compared to WT neurons with GOrilla tool by molecular function.**

Figure S5. **Calcium entry evoked by store depletion is significantly increased in HD iPSCs derived neurons.**

Figure S6. **NF-κB activity in PSCs derived neurons.**

Table S1. **Differentially expressed genes in HD iPSCs derived neurons.**

Supplemental Methods


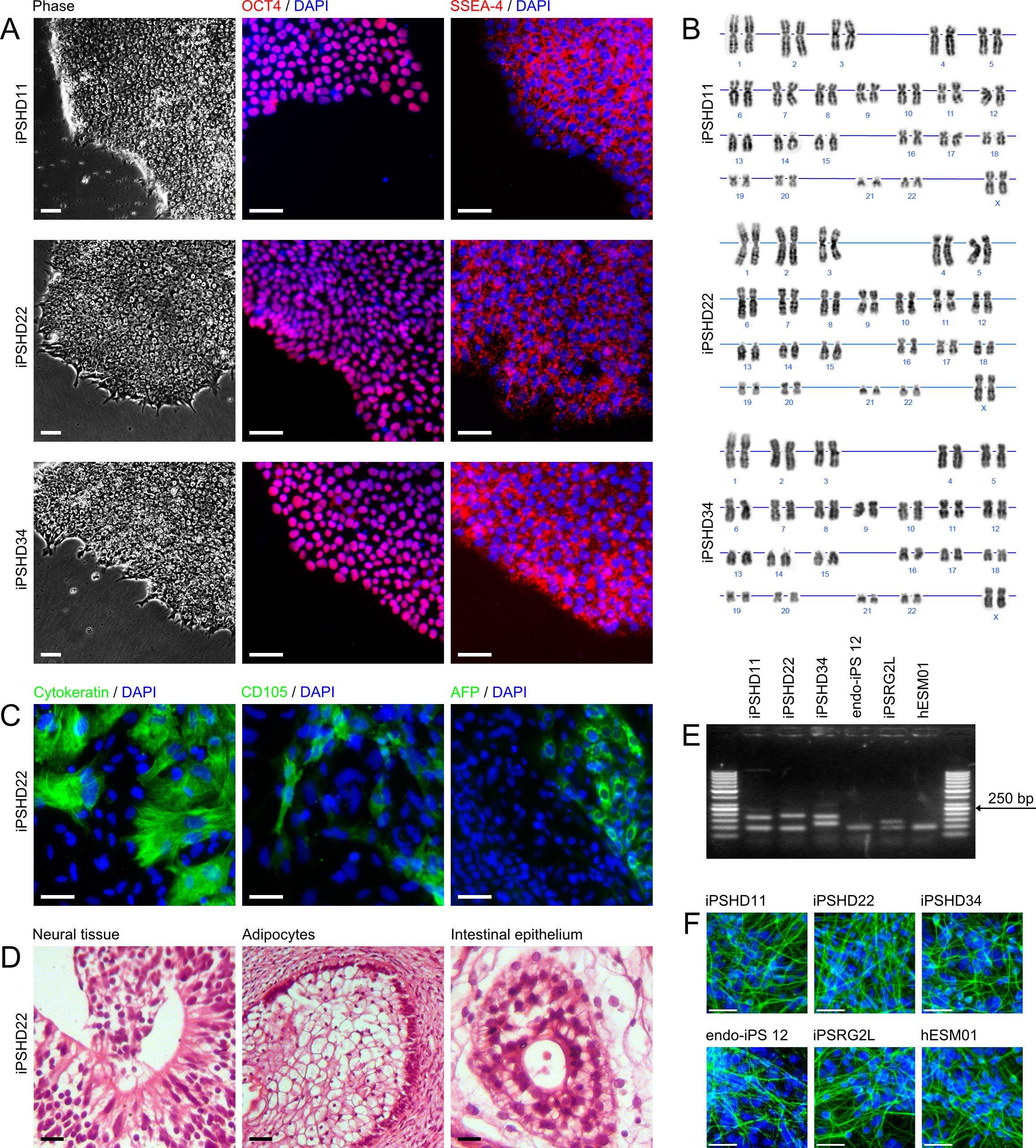


**Figure S1 PSC lines characterization.** **(A)** Phase contrast and immunohistochemical analysis for OCT4 (red) and SSEA-4 (red) expression in HD iPSCs, nuclei are counterstained with DAPI (blue), scale bar 50 μm. **(B)** Karyotype of HD iPSC lines by GTG-banding. **(C)** Representative images of spontaneously differentiated iPSHD22 cell line immunostained for ectoderm (pan-cytokeratin, red), mesoderm (CD105, red), and endoderm (α-fetoprotein, red) markers, nuclei are counterstained with DAPI (blue), scale bar 50 μm. **(D)** Representative images of histological analysis of iPSHD22 derived teratomas stained with hematoxylin and eosin. Scale bar 50 μm. **(E)** PCR analysis of the CAG-repeat length in PSC lines. **(F)** Immunohistochemical analysis for TUBB3 (green) expression in neurons differentiated from PSC lines, nuclei are counterstained with DAPI (blue), scale bar 40 μm.


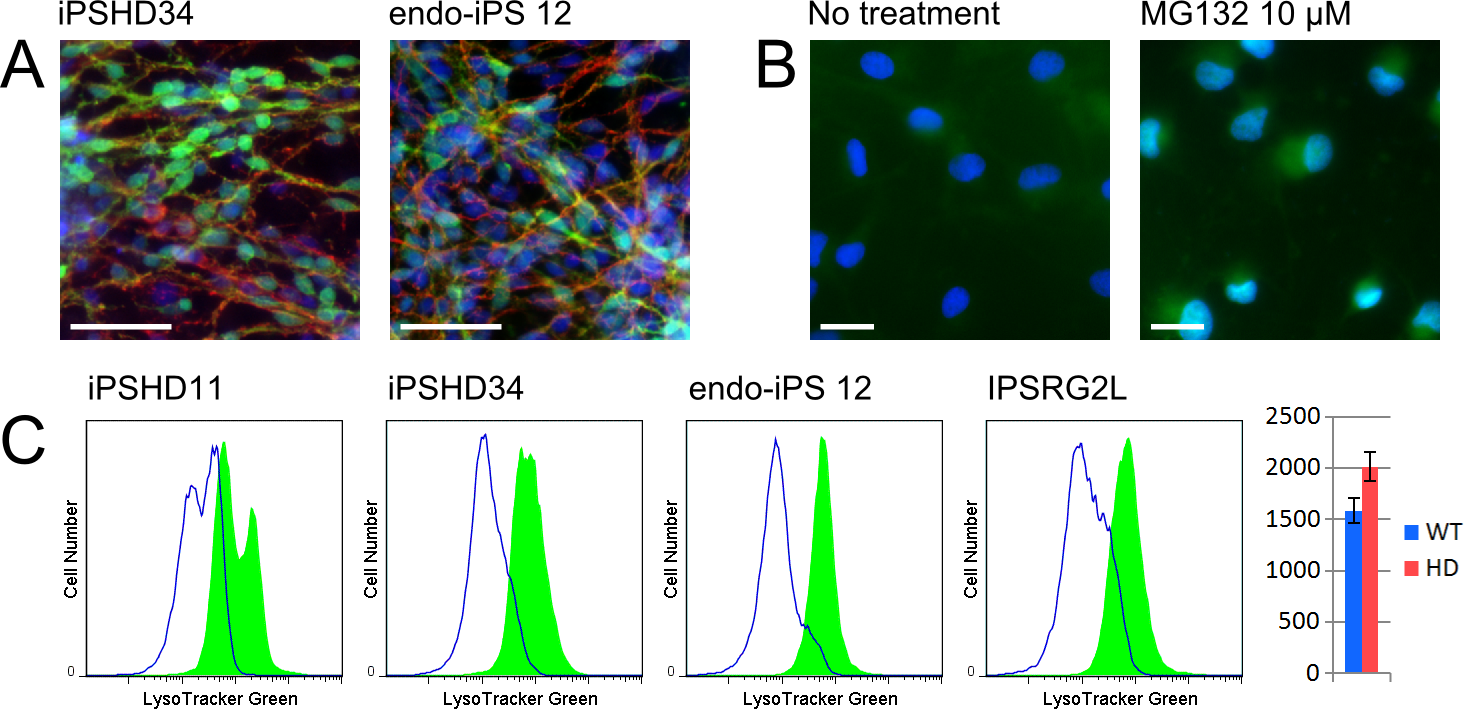


**Figure S2 HD and WT PSC derived neurons analysis.** **(A)** Representative images of HD and WT neurons immunostained for DARPP-32 (green) and TUBB3 (red) counterstained with DAPI (blue). Scale bar, 50 μm. **(B)** Neurons differentiated from endo-iPS12, immunostained using antibody EM48 (green), and counterstained with DAPI (blue) following a 24 h incubation with or without 10 μM MG132. Scale bar, 20 μm. **(C)** FC analysis of live neurons stained with LysoTracker Green (green) and without staining (blue). The barplot demonstrates median fluorescence intensity from these experiments, WT is mean ± SEM of endo-iPS12 and IPSRG2L while HD is mean ± SEM of iPSHD11 and iPSHD34. The scale on the y-axis is given in RFU.


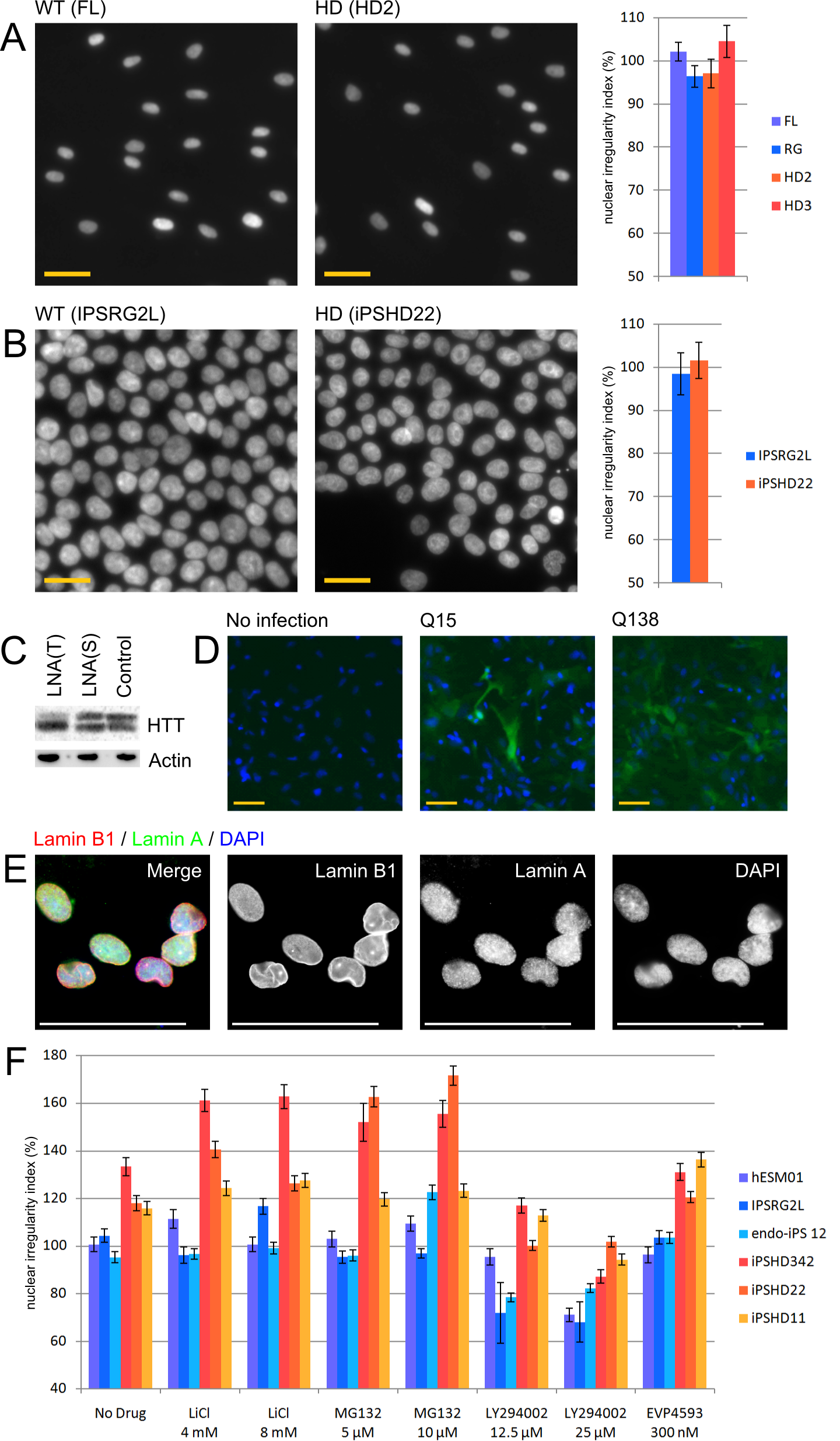


**Figure S3 Nuclear indentations in HD PSC derived neurons.** **(A)** Representative microphotographs of WT and HD fibroblasts stained with DAPI. Scale bar, 50 μm. The bar plot represents morphometric quantification of nuclear irregularity index in cultures of WT (FL, RG) and HD (HD2, HD3) fibroblasts using 327–676 nuclei per data point. **(B)** Representative microphotographs of WT and HD PSCs stained with DAPI. Scale bar, 50 μm. The bar plot represents morphometric quantification of nuclear irregularity index using 402–494 nuclei per data point. **(C)** Western blot analysis with antibodies for HTT (ab109115, Abcam) and Actin (A3854, Sigma-Aldrich) of neurons 4 days after transfection with antisense oligonucleotides: LNA(T) – allele-specific mHTT knockdown, LNA(S) – scrambled oligonucleotide, Control – no transfection. **(D)** Representative microphotographs of cells, infected with lentiviral vectors containing first exon of *HTT* with 15Q, first exon of *HTT* with 138Q and without infection, immunostained using EM48 antibody (green), costained with DAPI (blue). Microphotographs were captured using the same exposition time. Scale bar, 50 μm. **(E)** HD neurons immunostained for Lamin B1 and Lamin A, nuclei are counterstained with DAPI, scale bar 50 μm. **(F)** Mean nuclear irregularity index of HD and WT neurons treated with different drugs for 24 hours. The data are shown as mean ± SEM.


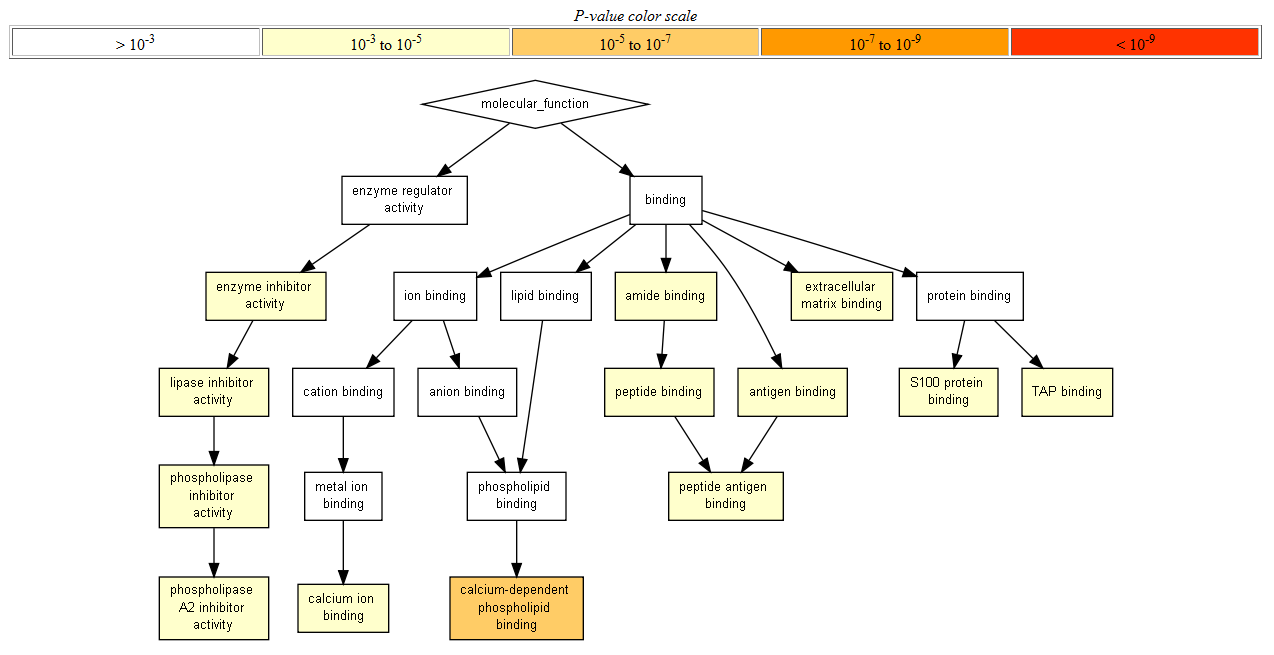


**Figure S4 Classification of up-regulated genes in HD neurons compared to WT neurons with GOrilla tool by molecular function.**


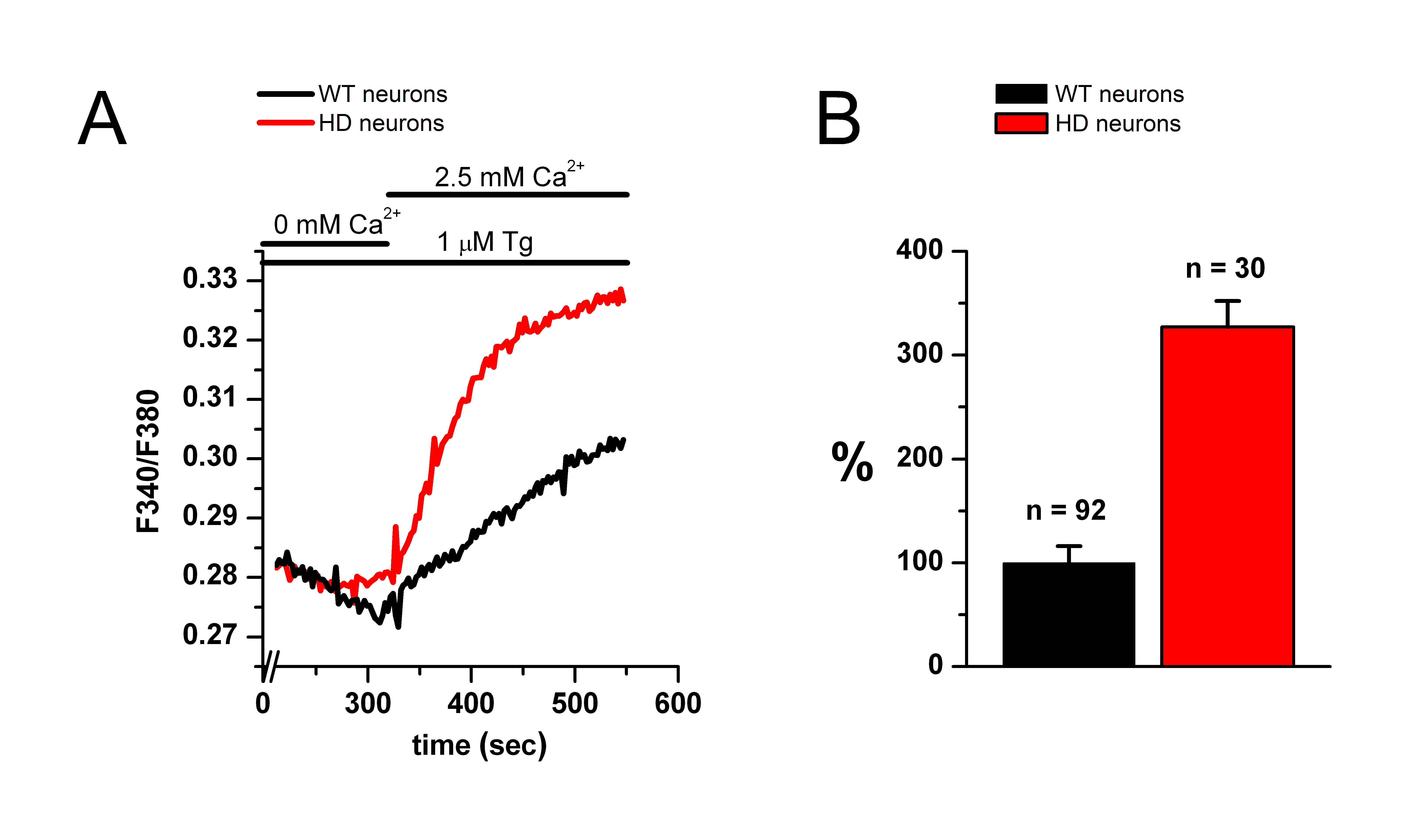


**Figure S5 Calcium entry evoked by store depletion was significantly increased in HD iPSCs derived neurons.** **(A)** Cytosolic Ca^2+^ levels in HD neurons (red line) and WT Neurons (black line) were monitored by ratiometric Fura-2 imaging. Horizontal lines on the top indicate the time of application of medium respectively containing: 0 mM Ca^2+^and 0.2 mM EGTA; 2.5 mM Ca^2+^; 1µM Tg. Each trace demonstrates a characteristic individual experiment. **(B)** The amount of calcium entry evoked by store depletion in WT Neurons (black) and HD Neurons (red) neurons. The results measured for 92 and 30 cells respectively and plotted as a percentage of the control amount. The data are shown as mean ± SEM.


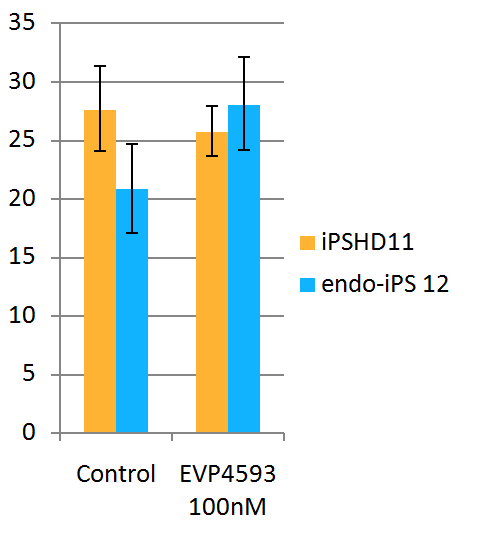


**Figure S6. NF-κB activity in PSCs derived neurons.** Mean normalized NF-κB reporter activity in iPSHD11 and endo-iPS12 derived neurons after EVP4593 treatment.

**Table S1 Differentially expressed genes in HD iPSCs derived neurons**.

| **Expression in HD Neurons** | **Genes** |
| --- | --- |
| Enhanced | ACPL2, ACTN1, ALDH3A2, ANGPTL4, ANXA1, ANXA2, ANXA2P1, ANXA5, ANXA7, ARHGEF3, ARHGEF6, ARMCX3, ASAP1, B2M, C11orf46, C11orf67, C17orf79, C1orf93, C5orf32, CA12, CADM1, CANX, CAPZA2, CCDC28A, CCND3, CHST15, CHURC1, CMPK1, COL4A1, COL4A5, COX17, CPNE3, CREG1, CXorf57, CYBRD1, DLK1, DPY19L1, DSTN, DYRK4, EFR3A, ELK1, ELOVL5, EMP1, EPDR1, EPHB1, FABP5, FABP5L2, FAM120A, FAM127C, FAM190B, FCGRT, FERMT2, FGFRL1, FHL1, FLJ10916, FLJ14213, FNDC3B, FOXO1, GAD1, GATM, GJA1, GLCE, GPRASP2, HDAC4, HLA-A, HLA-A29.1, HLA-F, HLA-G, HLA-H, HSD17B10, HSD17B12, IER3, IFI27L1, IFI27L2, IFT57, IGFBP7, IRS2, ITPRIP, KBTBD2, KIAA0182, KIAA0494, KLHL13, LAMA5, LAMC1, LAMP2, LANCL1, LGALS1, LHFP, LIMA1, LIPG, LMNA, LOC100134073, LOC100134361, LOC641768, LOC647307, LOC730278, LYPD1, MAOA, MAPK4, MBIP, MBNL2, MED14, METTL7A, MGMT, MGST1, MID1IP1, MT1A, MT1F, MT1X, MT2A, MTMR11, MX1, MXRA7, MYH9, NFE2L2, NPAL3, NQO1, OCIAD2, OLFML2A, OLIG1, OLIG2, PALLD, PCP4, PCSK1, PDGFRB, PHF11, PHYH, PLOD2, PLOD3, PNPO, PPP1R3C, PROS1, PURB, RAB5B, RBL2, RBM23, RGL1, RGS2, RHOB, RIPK5, S100A11, S100A6, SCG5, SELM, SERPINH1, SH3BP4, SIDT2, SLC2A10, SLC2A12, SLC9A3R1, SOX8, SPARC, SPARCL1, SPATS2L, SPIRE1, SPOCK2, SQSTM1, SSR1, STAG2, STOM, TACC1, TAP1, TCEAL4, TIMP1, TIPARP, TMEM43, TMEM47, TMEM50B, TMSB4X, TPM2, TPP1, TSKU, TSPAN7, TSPAN9, TYK2, VAMP3, VAMP5, VASN, WDR1, ZBTB20, ZFAND6, ZMAT3, ZSCAN18. |
| Reduced | ABLIM1, ASNS, AUTS2, BTBD17, C16orf48, C8orf13, CCDC23, CENPV, CPXM1, DNLZ, ELAVL3, ELAVL4, EVL, FLJ22795, FLJ25404, GPC2, H2AFY2, HNRNPAB, KLHL35, LOC347544, LOC388556, LOC644919, LOC647000, LOC650757, LOC653344, LOC729679, LOC92755, LRRN1, MAP6, MDK, MGC61598, NEFM, NELL2, NR2F1, NR2F2, PGRMC1, REM2, RNASEH2A, RPL39L, RPL9, SALL2, SIPA1L2, SMA4, STMN2, TUBB, TUBB2A, TUBB3, TUBB4, TUBB4Q, U2AF1, WDR68, ZIC2. |

**Supplemental Methods**

*Culture and isolation of fibroblasts from human skin biopsies*

After signing the informed consent patients underwent skin biopsy of the forearm. Biopsy was placed in a drop of medium on Petri dish and cut into small pieces (the size of about 1 mm^3^) with sharp sterile scalpel. The resulting pieces were placed in separate 35 mm Petri dishes in 3 ml of culture medium and pressed with sterile coverslip (MENZEL-GLASER, Germany). Medium was changed once a week. After 3 weeks fibroblasts were detached and passaged. Fibroblasts were grown in DMEM (PanEco, Russia), 15% Fetal Bovine Serum (FBS) (Hyclone, USA), penicillin-streptomycin (PanEco, Russia). The medium was changed every 2-4 days. Cells were passaged using 0,25 % trypsin (Hyclone, USA) or cryopreserved with FBS (Hyclone, USA) supplemented with 10% DMSO (PanEco, Russia).

*Lentiviral vectors production and application*

Lentiviral particles were assembled according to [1]. Viral titers were determined by immunohistochemistry of HEK293 cells, infected with sequential viral dilutions using antibodies to the relevant antigen. To infect cells Polybrene 8 mg/ml (Sigma-Aldrich, USA) was added one hour prior lentiviral transduction.

*Generation of iPSCs*

5x10^4^ human skin fibroblasts were plated to 35 mm culture dish (Corning, USA) in the culture medium for fibroblasts supplemented with 2 ng/ml bFGF (PeproTech, USA). Two days after the plating, cells were transduced with lentiviral vectors LeGO-hOCT4, LeGO-hSOX2, LeGO-hc-Myc, LeGO-hKLF4. During the first week after transduction valproic acid (VPA) and BIX-01294 (Sigma-Aldrich, USA) were added in culture medium to the final concentration 1 mM and 2 mM respectively. 5 days after transduction, cells were replated 1:12 in a new dish. The next day after replanting culture medium was changed to ESC medium: DMEM/F12 (Hyclone, USA), 20% KO Serum replacement (Invitrogen, USA), 0.1mM β-mercaptoethanol (Sigma-Aldrich, USA), 1% NEAA (Hyclone, USA), bFGF 4 ng/ml (PeproTech, USA), 50 units/mL penicillin, and 50 μg/mL streptomycin (PanEco, Russia). Cells were cultured in this medium for 10-12 days. Individual colonies were mechanically picked and cultured separately in mTeSR1 (Stem Cell Technologies, Canada) on Matrigel (BD Biosciences, USA).

*Formation and culture of embryoid bodies*

For embryoid bodies formation PSC colonies were detached with dispase 1 mg/ml (Invitrogen, USA), dissociated into fragments of 400-600 cells, transferred to Ultra Low Adhesion Plates (Corning, USA) and cultured in DMEM/F12 (Hyclone, USA), 20% FBS (Hyclone, USA) , 0.1mM β-mercaptoethanol (Sigma-Aldrich, USA) , 1% NEAA (Hyclone, USA), penicillin-streptomycin (PanEco, Russia). Medium was changed every 2 days.

*Spontaneous differentiation of human PSCs*

Spontaneous differentiation of PSCs was performed through embryoid body formation. Embryoid bodies were grown for 10-20 days, plated to gelatin-coated Petri dishes (Corning, USA), after that cultured for 15-20 days in the medium for embryoid bodies.

*Teratoma formation*

Before injections dishes with PSCs were washed with DMEM/F12 (PanEco, Russia), treated with dispase (Invitrogen, USA) for 6 min at 37 °C, cells were scraped and precipitated by centrifugation. Collected cells were washed out of enzyme with DMEM/F12 (PanEco, Russia) and mixed with Matrigel (BD Biosciences) on ice. Approximately 5×10^6^ cells were subcutaneously injected in hind leg of *nude/nude* mice. 10-14weeks after injection teratomas were dissected, rinsed with PBS (PanEco, Russia), and fixed in Bouin's Fixative. Paraffin sections were stained with Hematoxylin/Eosin and histological analysis of tissues was performed.

*Culture media for neuronal differentiation*

The components for preparation of neuronal differentiation media were as follows: DMEM/F12 (PanEco, Russia), Neurobasal-A (Life Technologies, USA), N2 (Life Technologies, USA), B27 (Life Technologies, USA), recombinant human Noggin (PeproTech, USA), recombinant human bFGF (PeproTech, USA), recombinant human BDNF (PeproTech, USA), SB431542 (Stemgent, USA), Dorsomorphin (Stemgent, USA), Purmorphamine (Stemgent, USA), Forskolin (Stemgent, USA). Four culture media were used to differentiate PSCs into neurons: K-1 medium: DMEM/F12, N2 1x, Noggin 80 ng/ml, bFGF 4 ng/ml, SB431542 8 μM, Dorsomorphin 2 μM; K-2 medium: DMEM/F12, N2 1x, Noggin 80 ng/ml, bFGF 4 ng/ml, Purmorphamine 0.65 μM, K-3: DMEM/F12, N2 1x, bFGF 10 ng/ml, Purmorphamine 0.65 μM, Forskolin 4 μM, K-4: Neurobasal-A, B27 1x, BDNF 10 ng/ml, Forskolin 4 μM.

*Flow cytometry analysis of* LysoTracker® Green *stained neurons*

Neurons were cultured in 48 well plate in K-4 medium On the day of analysis neurons were detached with TrypLE™ Express (Life Technologies, USA), suspended with pipetting, TrypLE™ Express was inactivated by medium (DMEM/F12, FBS 10%). Collected cells were pelleted at 400 g, resuspended in DMEM/F12 to concentration of 2×10^6^ cells/ml, incubated for 5 min at 37 °C and treated with LysoTracker® Green DND-26 (Life Technologies, USA) at final concentration of 75 nM for 30 min at 37°C in the dark. After washing in ice-cold Ca/Mg free PBS with 0.1% BSA cells were treated with CD56-PE / IgG-PE antibody 1.5ug/ml (Supplemental table 1) for 40 min on ice. Stained cells were then washed twice, resuspended in ice-cold Ca/Mg free PBS with 0.1% BSA to concentration of 2×10^6^ cells/ml and subjected to fluorescent activated cell analysis on Gallios Flow Cytometer (450-nm, 488-nm lasers) (Beckman Coulter, USA). Dead cells were gated out by DAPI (Sigma-Aldrich, USA) 0.2 μg/ml (450 nm filter) and Propidium Iodide (Becton Dickenson, USA) 1 μg/ml (695 nm filter) incorporation. Therefore, only live NCAM-positive neurons (CD56-PE, 575nm filter, FL2/FL4 compensation) were accounted in LysoTracker® Green Mean Florescence Analysis (525 nm filter).

*Fura-2 Ca^2+^ imaging*

Neurons grown on glass coverslips were loaded with 5 µM Fura-2AM in the presence of 0.025% Pluronic for 40 min at 37°C. Loaded cells were illuminated by alternating 340 and 380-nm excitation light at 2 Hz. Emission fluorescence intensity was measured at 510 nm with the use of an InCyt Basic I/P dual wavelength fluorescence imaging system (Intracellular Imaging Inc., Cincinnati, OH). The change in cytosolic Ca^2+^ concentration was expressed as the ratio of emission fluorescence intensity at 340 and 380 nm excitation wavelengths (340/380 ratio). Store-operated Ca^2+^ entry was evoked by application of 1 µM Tg. Depolarization of membrane was evoked by application of 130 mM KCl.

*Immunocytochemistry*

Cells were washed 2 times with PBS (PanEco, Russia) and fixed with 4% paraformaldehyde (Sigma-Aldrich, USA) for 20 min at room temperature, then washed with PBS-0.1% Tween20 (Sigma-Aldrich, USA) 3 times. Non-specific absorption of antibodies was blocked by incubation for 30 min in PBS containing 0.1% Tween20, 5% FBS (Hyclone, USA), 2% goat serum (Hyclone, USA), and 0.1% Triton X-100 (Sigma-Aldrich, USA) at room temperature. Primary antibodies were applied at dilutions recommended by the manufacturer in PBS containing 0.1% Tween20, 5% FBS, and 2% goat serum, incubated for 1 hour at room temperature, and washed 3 times for 5 min in PBS-0.1% Tween20. Secondary antibodies were applied at dilutions recommended by the manufacturer, incubated for 30 min at room temperature in the dark, washed 3 times for 5 min in PBS-0.1% Tween20, incubated with DAPI (4',6-diamino-2-feniliindol dihydrochloride) (Sigma-Aldrich, USA) 0.1 μg/ml in PBS for 10 minutes, and washed 2 times in PBS-0.1% Tween20. Antibodies used are listed below. Images were acquired with fluorescent microscope Axiovert 40 CFL (Zeiss AG, Germany) or Axio Imager A1 (Zeiss AG, Germany) and processed with Axiovision software.

Table of antibodies used in the study.

| **Gene** | **Cat. No.** | **Manufacturer** |
| --- | --- | --- |
| Lamin B1 | ab16048 | Abcam |
| Lamin A | ab8980 | Abcam |
| β-III-Tubulin | ab18207 | Abcam |
| β-III-Tubulin | ab7751 | Abcam |
| DARPP-32 | ab40801 | Abcam |
| HTT | ab109115 | Abcam |
| GAT-1 | ab64645 | Abcam |
| MAP2 | ab5392 | Abcam |
| PAX6 | ab78545 | Abcam |
| NCAM1 | ab6123 | Abcam |
| FOXP2 | ab16046 | Abcam |
| ENO2 | ab79757 | Abcam |
| OCT4 | ab18976 | Abcam |
| Сytokeratin(PAN) | M0821 | DAKO |
| CD105 | M3527 | DAKO |
| AFP | A0008 | DAKO |
| CD56 | R7251 | DAKO |
| IgG2b | X0951 | DAKO |
| SSEA-4 | MC-813-70 | DSHB |
| SOX2 | cell signalling 3579 | Cell Signaling Technology |
| Nestin | MAB5326 | Millipore |
| EM48 | MAB5374 | Millipore |

*Quantification of DARPP-32 positive cells*

Differentiated neurons were immunostained with antibodies to DARPP-32 and TUBB3 and costained with DAPI. Random microscopy fields were captured with fluorescent microscope Axiovert 40 CFL (Zeiss AG, Germany). Areas where cells were grown in a single layer were used for quantification by manual counting. The cells were identified by DAPI stain, the number of neurons was determined by TUBB3 staining, the number of DARPP-32+ neurons was determined by dual TUBB3 and DARPP-32 staining. Approximately 1000 cells in four independently differentiated cell lines were analyzed.

*SDS-PAGE and Western Blotting Analysis*

Cells were lysed in RIPA lysis buffer (50 mM Tris, pH 7-8, 150 mM NaCl, 0.1% SDS, 0.5% sodium deoxycholate, 1% Triton X-100). Lysates of 100000 cells were diluted in 4x Laemmli sample buffer (250 mM Tris-HCl pH 6.8, 8% SDS, 40% glycerol, 8% 2-mercaptoethanol, 0.02% bromophenol blue), incubated at 95°C for 5 minutes and spun down at 12000 g before loading. To separate WT HTT (~350 kDa) and mutant HTT (~353 kDa), proteins in cell lysates were separated by SDS-PAGE in a large PerfectBlue Dual Gel System electrophoresis unit (Peqlab, Germany). Gels (200×200×1.5 mm) consisted of a 7% resolving gel (acrylamide:bisacrylamide 37.5:1, 450 mM Tris-acetate pH 8.8, 0.1% SDS, 0.1% ammonium persulfate and 0.015% TEMED) coupled to a 4% stacking gel (acrylamide:bisacrylamide 37.5:1, 150 mM Tris-acetate pH 6.8, 0.1% SDS, 0.1% ammonium persulfate and 0.015% TEMED). Gels were run at 200 mA per gel, up to 400 V for 6 h in Tris-acetate SDS buffer (50 mM Tricine, 50 mM Tris base, 0.1% SDS, pH 8.24) cooled by water. PageRuler™ Plus Prestained Protein Ladder (Thermo Scientific, USA) was used to control electrophoresis. To perform Western blot loading control, cell lysates were analyzed for β-actin expression. Laemmli SDS–PAGE [2] was performed using 12% resolving gel, coupled to 5% stacking gel. Gels were run at 35 mA, up to 240 V for 60 min in Tris-glycine buffer (25 mM Tris, 250 mM glycine, 0.1% SDS). After separation, a gel piece above the 250 kDa ladder band, was cut out and used for further analysis. The separated proteins were transferred to a PVDF membrane using Trans-Blot® Turbo™ Transfer System (Bio-Rad, USA) with Trans-Blot® Turbo™ Midi PVDF Transfer Packs (Bio-Rad, USA). Electrophoretic transfer was performed at 2.5 A, up to 25 V for 10 min. After blotting, PVDF membrane was rinsed in deionized water and incubated in phosphate-buffered saline with 0.1% Tween20 (PBST) containing 3% BSA for 1 h to block nonspecific binding. Membrane was treated with rabbit anti-HTT antibodies (ab109115, Abcam, USA) at a 1:5000 dilution. After overnight incubation at +4°C, membrane was washed three times with PBST. Then, membrane was treated with anti-rabbit IgG conjugated with horseradish peroxidase (GE Healthcare, USA) at a dilution of 1:10000 in PBST for 2 h at room temperature. For Western blot loading control, the proteins were transferred (semi-dry transfer, 1 mA/cm2 for 1 h) to a PVDF membrane (GE Healthcare, USA) in Tris-glycine transfer buffer (48 mM Tris, 39 mM glycine, 0.04% SDS, 20% methanol). Then, membrane was rinsed in deionized water and incubated in PBST containing 3% BSA for 1 h. Further, membrane was treated with anti-actin antibodies conjugated with HRP (A3854, Sigma-Aldrich, USA), at a 1:20000 dilution overnight at +4°C. After a final wash, membranes were treated with ECL Plus Western blotting detection reagents (GE Healthcare, USA) according to the manufacturer recommendations. The signals were detected using the ChemiDoc™ XRS+ System (Bio-Rad, USA).

*NF-κB analysis*

Cells were cultured in K-4 medium in a 96-well black plates with clear flat bottom (Corning, USA). Cells were infected with pHAGE NFkB-TA-LUC-UBC-GFP-W lentiviral reporter construct described earlier [4]. Three days after transduction, cells were treated with chemical compounds for 6 h prior to analysis. Luminescent assay Dual-Luciferase® Reporter Assay System (Promega, USA) was used to measure luciferase activity in each well. Measurements were performed according to the manufacturer’s instructions. Luminescence and GFP fluorescence to normalize transduction efficacy were detected using DTX 880 Multimode Microplate Reader (Beckman Coulter, USA).

*RT-PCR analysis*

Total RNA from cell cultures was isolated using RNAeasy Mini kit (Qiagen, USA) according to the manufacturer's instructions. DNAse digestion was carried out directly on the columns. Reverse transcription was performed with random hexamer primers (Evrogen, Russia), M-MLV Reverse Transcriptase (Promega, USA), Ribonuclease Inhibitor (Promega, USA), dNTPs (Thermo scientific, USA) according to the manufacturer's instructions. 0.5-1 mg of total RNA has been used for reaction. PCR amplification was performed using ScreenMix (Evrogen, Russia) according to manufacturer's instructions. The list of primers used is shown in the table below.

Table of primers used in the study.

| **Gene** | **Primers sequence** | **Product size** |
| --- | --- | --- |
| GAPDH | 5’TTTTGCGTCGCCAGCCGAG3’  5’TAAGCAGTTGGTGGTGCAGGAGGC3’ | 508bp |
| PPP1R1B | 5’AGATCCGGCGCAGGAGACCAACG3’  5’CCCTAGCAGGCGAGGGGAAGAG3’ | 642bp |
| GAD1 | 5’GATATTTTCTCCTGGGGGCG3’  5’GGCCACACTGAATTGCCTTG3’ | 632bp |
| DRD1 | 5’CAGGGGCTTTGAGAGAGACG3’  5’AGCAGGGAATAGGGGTCAGT3’ | 467bp |
| BCL11B | 5’ATGCCAGAATAGATGCCGGG3’  5’ATGTTCTCCTGCTTGGGACAG3’ | 445bp |
| CALB1 | 5’GCAAACAAGACTGTTGATGACAC3’  5’AAGAGCAAGATCCGTTCGGTAC3’ | 363bp |
| SST | 5’GCTTTAGGAGCGAGGTTCGG3’  5’CATTCTCCGTCTGGTTGGGT3’ | 292bp |
| RASD2 | 5’GATACCTCTGGCAACCACCC3’  5’AAGTAGGCGCAGTTCTCGTC3’ | 290bp |
| PENK | 5’TGACACTTTGCACTTGGCTG3’  5’TGGCTTTCTTCCGGTTTGCT3’ | 268bp |
| ANO3 | 5’GGCCCAAAACCCAATGGTTC3’  5’TGTGGTGGAAACGCTGCTAT3’ | 255bp |
| PDYN | 5’TCATGTTCCCCTCCACCACA3’  5’CTTGGCCAGCTCACTGTAGG3’ | 254bp |
| TRPC1 | 5’CCTACACTGGTGGCAGAAGG3’  5’GCAAAGCAGGTGCCAATGAA3’ | 299bp |
| TRPC3 | 5’CAGGGTGAAAACCACCCAGT3’  5’TGAGCACAACAGCTATGGCA3’ | 365bp |
| GRIA1 | 5’GAAGGTGATGGCTGAGGCTT3’  5’TCGGATGCCGTCATGTTTCA3’ | 241bp |
| GRIA2 | 5’CCTTTATGCGGCAAGGATGC3’  5’GGCCGTAGTCCTCACAAACA3’ | 314bp |
| GRIK2 | 5’CACCTCCGAAACCCGATTCA3’  5’CTGTGAGGCCTTCCCAATGT3’ | 210bp |
| GRIK5 | 5’TGCCAGGTGCTCTCATCAC3’  5’CAGATGCTGGGCTAGAGGAG3’ | 256bp |
| GRIN1 | 5’CAAGTATGCGGATGGGGTGA3’  5’CTTGACGTACACGAAGGGCT3’ | 253bp |
| GRIN2B | 5’TCACTCCCTTAATCTGTCCGTC3’  5’ACATGAGATCACAGATGCGGG3’ | 334bp |
| OCT4 | 5’CGACCATCTGCCGCTTTGAG3’  5’CCCCCTGTCCCCCATTCCTA3’ | 573bp |
| SOX2 | 5’TCCTGATTCCAGTTTGCCTC3’  5’GCTTAGCCTCGTCGATGAAC3’ | 480bp |
| c-Myc | 5’AGTAATTCCAGCGAGAGGCA3’  5’AGGCTGCTGGTTTTCCACTA3’ | 389bp |
| NANOG | 5’CAGCCCTGATTCTTCCACCAGTCCC3’  5’TGGAAGGTTCCCAGTCGGGTTCACC3’ | 391bp |
| FOXD3 | 5’CAAGCCCAAGAACAGCCTAGTGAA3’  5’TGACGAAGCAGTCGTTGAGTGAGA3’ | 203bp |
| SALL4 | 5’TGTGACTTTACGGGTTCTGAGCCA3’  5’TGTACTGGTTCCACACAACAGGGT3’ | 931bp |
| HTT | 5’CCTTCGAGTCCCTCAAGTCCTTC3’  5’GGCTGAGGAAGCTGAGGAG3’ | vary |
| GSX2 | 5’AAGAGCCAGTTCTCTTCGGC3’  5’AATCCTCCGGAGTCGAGACA3’ | 384 |
| PAX6 | 5’GTCAGGCTTCGCTAATGGGC3’  5’GATTCCACGGGGCTCGAATA3’ | 390 |
| FOXG1 | 5’GGCAAGGGCAACTACTGGAT3’  5’CTGAGTCAACACGGAGCTGT3’ | 294 |
| OTX2 | 5’AAACCAGCCCCTCTGTTTGTT3’  5’GCGGCACTTAGCTCTTCGAT3’ | 440 |

*HPLC analysis for amino acids secretion*

K-4 culture medium was replaced by DMEM/F12. Neurons were incubated for 30 min at 37 °C in DMEM/F12. Then supernatant was collected and used for HPLC. HPLC analysis of supernatant was performed as a standard medical test for 32 amino acids by Chromolab LTD (Russia).

*Microarray gene expression analysis*

Microarray experiments were conducted using HumanHT-12 v4 Expression BeadChip (Illumina, USA) in two technical repeats for each sample. Bioinformatic analysis was performed using GenomeStudio (Illumina, USA). Differentially expressed genes were found for each pair of differentiated in parallel samples (HD and WT). Normalization was performed by averaging without background subtraction. Genes considered differentially expressed, under the conditions of DiffScore > 25 and p < 0.05 for detection in all samples. Genes that were differentially expressed in all pairs were selected for further analysis (Supplemental table 1). Enrichment analysis was performed using online instrument <http://cbl-gorilla.cs.technion.ac.il/> in two unranked lists of genes mode.

*Chemicals*

The following substances in the given final concentrations were used: 4-8 mM LiCl (Sigma-Aldrich, USA), 5-10 μM MG132 (Abcam, UK), 12.5-25 μM LY294002 (Tocris Bioscience, UK), 250-1000 nM Thapsigargin (Tocris Bioscience, UK), 30-3000 nM EVP4593 (Sigma-Aldrich, USA), 100 μM DTT (Life Technologies, USA), 50 μM 2-APB (Sigma-Aldrich, USA), 2 μM Ruthenium Red (Tocris Bioscience, UK). Solvents without substance were used as a control.

*Selection of striatal-specific markers*

Neurons specific genes were selected as the genes that were specific for striatum with online resource <http://www.brain-map.org/>. Selected genes were validated with another online resource <http://amazonia.transcriptome.eu/>.

*Electron microscopy*

Cells were grown on the special plastic film (Agar Scientific Ltd., UK) 2x2 sm^2^ in K-4 medium. Cells were fixed with 2.5 % (w/v) glutaraldehyde in culture medium for 15 min, then with 2.5 % (w/v) glutaraldehyde in 0.1 mol/L sodium cacodylate buffer (pH 7.2) for 1 hour. This was followed by three washes in the same buffer for 5 min each and postfixation in 1 % (w/v) OsO_4_ and 0.8 % (w/v) potassium ferrocyanide for 1 hour. After three washes in mQ water samples were placed in a 1 % aqueous solution of uranyl acetate (Serva, Heidelberg, Germany) for 1 hour at room temperature. After that the samples were dehydrated in an ethanol series (30 %, 50 %, 70 %, 96 % for 10 min, and 100 % for 20 min) and acetone (twice, for 20 min), and embedded in Agar 100 Resin (Agar Scientific Ltd., UK). Semi-thin sections were obtained with a Reichert-Jung ultracut microtome, stained with methylene blue and analyzed with an Аxioscop 40 (Zeiss) light microscope. Ultra-thin sections were made using a Leica ultracut ultra-microtome and stained with Reynolds lead citrate. Sections were examined with a transmission electron microscope JEM-100SX (JEOL) at the Centre for Microscopic Analysis of Biological Objects, IC&G SB RAS. To analyze the irregularity of nuclei the percent of normal nuclei or nuclei with deep indentations of nuclear membrane have been calculated on thin sections by TEM. Nuclei from 57 and 113 cells on randomly chosen 5 sections of iPSHD22 and hESM01 samples have been analyzed correspondently. To analyze autophagy on sections by TEM cells of iPSHD22 and hESM01 containing nucleus and integrity of the plasma membrane were randomly selected, the number autophagic vacuoles per 1 μm^2^ of cell cytoplasm was calculated using ImageJ.

**References**

1. Weber K, Mock U, Petrowitz B, Bartsch U, Fehse B: **Lentiviral gene ontology (LeGO) vectors equipped with novel drug-selectable fluorescent proteins: new building blocks for cell marking and multi-gene analysis.** *Gene Ther* 2010, **17**:511–20.

2. Laemmli UK: **Cleavage of structural proteins during the assembly of the head of bacteriophage T4.** *Nature* 1970, **227**:680–5.

3. Gagnon KT, Pendergraff HM, Deleavey GF, Swayze EE, Potier P, Randolph J, Roesch EB, Chattopadhyaya J, Damha MJ, Bennett CF, Montaillier C, Lemaitre M, Corey DR: **Allele-selective inhibition of mutant huntingtin expression with antisense oligonucleotides targeting the expanded CAG repeat.** *Biochemistry* 2010, **49**:10166–78.

4. Wilson AA, Kwok LW, Porter EL, Payne JG, McElroy GS, Ohle SJ, Greenhill SR, Blahna MT, Yamamoto K, Jean JC, Mizgerd JP, Kotton DN: **Lentiviral delivery of RNAi for in vivo lineage-specific modulation of gene expression in mouse lung macrophages.** *Mol Ther* 2013, **21**:825–33.
